# *ADCK2* Haploinsufficiency Reduces Mitochondrial Lipid Oxidation and Causes Myopathy Associated with CoQ Deficiency

**DOI:** 10.3390/jcm8091374

**Published:** 2019-09-02

**Authors:** Luis Vázquez-Fonseca, Jochen Schäefer, Ignacio Navas-Enamorado, Carlos Santos-Ocaña, Juan D. Hernández-Camacho, Ignacio Guerra, María V. Cascajo, Ana Sánchez-Cuesta, Zoltan Horvath, Emilio Siendones, Cristina Jou, Mercedes Casado, Purificación Gutierrez-Rios, Gloria Brea-Calvo, Guillermo López-Lluch, Daniel J.M. Fernández-Ayala, Ana B. Cortés, Juan C. Rodríguez-Aguilera, Cristiane Matté, Antonia Ribes, Sandra Y. Prieto-Soler, Eduardo Dominguez-del-Toro, Andrea di Francesco, Miguel A. Aon, Michel Bernier, Leonardo Salviati, Rafael Artuch, Rafael de Cabo, Sandra Jackson, Plácido Navas

**Affiliations:** 1 Centro Andaluz de Biología del Desarrollo, Universidad Pablo de Olavide-CSIC-JA, 41013 Sevilla, Spain; 2 Clinical Genetics Unit, Department of Women and Children’s Health, University of Padova, and IRP Città della Speranza, 35100 Padova, Italy; 3 Department of Neurology, Carl Gustav Carus University Dresden, 01307 Dresden, Germany; 4 Boston University School of Medicine, Boston, MA 02118, USA; 5 Translational Gerontology Branch, National Institute on Aging, National Institutes of Health, 251 Bayview Boulevard, Suite 100, Baltimore, MD 20201, USA; 6 CIBERER, Instituto de Salud Carlos III, 28000 Madrid, Spain; 7 Clinical Chemistry and Pathology Departments, Institut de Recerca Sant Joan de Déu, 08000 Barcelona, Spain; 8 Departamento de Bioquímica, Instituto de Ciências Básicas da Saúde, Universidade Federal do Rio Grande do Sul. CEP 90035-003 Porto Alegre, RS, Brazil; 9 Secciód’Errors Congènits del Metabolisme-IBC, Servei de Bioquímica I Genètica Molecular, Hospital Clinic, 08000 Barcelona, Spain; 10 División de Neurociencias, Universidad Pablo de Olavide, 41013 Sevilla, Spain

**Keywords:** Coenzyme Q deficiency, mitochondrial disease, respiratory chain, fatty acids, myopathy, aarF domain-containing mitochondrial protein kinase 2(ADCK2)

## Abstract

Fatty acids and glucose are the main bioenergetic substrates in mammals. Impairment of mitochondrial fatty acid oxidation causes mitochondrial myopathy leading to decreased physical performance. Here, we report that haploinsufficiency of *ADCK2*, a member of the aarF domain-containing mitochondrial protein kinase family, in human is associated with liver dysfunction and severe mitochondrial myopathy with lipid droplets in skeletal muscle. In order to better understand the etiology of this rare disorder, we generated a heterozygous *Adck2* knockout mouse model to perform in vivo and cellular studies using integrated analysis of physiological and omics data (transcriptomics–metabolomics). The data showed that *Adck2*+/− mice exhibited impaired fatty acid oxidation, liver dysfunction, and mitochondrial myopathy in skeletal muscle resulting in lower physical performance. Significant decrease in Coenzyme Q (CoQ) biosynthesis was observed and supplementation with CoQ partially rescued the phenotype both in the human subject and mouse model. These results indicate that ADCK2 is involved in organismal fatty acid metabolism and in CoQ biosynthesis in skeletal muscle. We propose that patients with isolated myopathies and myopathies involving lipid accumulation be tested for possible *ADCK2* defect as they are likely to be responsive to CoQ supplementation.

## 1. Introduction

The liver, adipose tissue, and skeletal muscle regulate systemic metabolic flexibility in order to maintain energy homeostasis during the fast–fed transition [1]. The ability to switch between glucose and fatty acid (FA) catabolism defines metabolic flexibility in times of energy abundance (e.g., feeding) and restriction. [2]. Mitochondria play a central role in cellular energy metabolism by providing reducing equivalents from the tricarboxylic acid cycle and FA β-oxidation to the respiratory chain (MRC). Mitochondrial respiration is directly involved in the ATP supply to match energy demand during excitation–contraction coupling in cardiac [3] and skeletal muscle [4]. Coenzyme Q (CoQ) is a fat-soluble compound that transfers electrons between complex I and II to complex III of the MRC, and also receives electrons from dihydroorotate dehydrogenase and sulfide: quinone oxidoreductase [5]. Mitochondria are the site for substrate selection, namely glucose or FA, depending upon energy demand [6] and CoQ contributes to determine the source of electrons from complex I (NADH) vs. complex II (FADH_2_) [7].

Defects in the mitochondrial β-oxidation pathway elicit clinically heterogeneous myopathies resulting from either multiple acyl-CoA dehydrogenase deficiency (MADD), an autosomal recessive disorder caused by mutations in *ETFDH*, a gene encoding electron-transfer flavoprotein: ubiquinone oxidoreductase, or mutations in *ETFA* or *ETFB*, which encode the alpha or beta subunit of electron transfer flavoprotein (ETF) [8,9]. Deficiency in ETF or the electron-transfer flavoprotein: ubiquinone oxidoreductase (ETFDH) leads to impaired FA oxidation and elevated levels of acyl-carnitines in plasma. The myopathic form of CoQ deficiency has been linked to a defect in FA oxidation in skeletal muscle [10,11,12]. Moreover, impaired lipid metabolism associated with aberrant mitochondrial morphology have been found in response to the loss of mitofusin 2 (Mfn2), resulting in MRC defects due to a secondary deficiency in CoQ [13]. Mitochondrial diseases associated with CoQ deficiency are rare and exhibit clinical heterogeneity that can be caused by mutations in *COQ* genes or genes encoded by either nuclear DNA or mitochondrial DNA [14,15,16,17].

The *ADCK* genes encode the aarF domain-containing mitochondrial protein kinases (ADCK1-5). Both human *ADCK3* (*COQ8A*) and *ADCK4* (*COQ8B*) show high homology to yeast *coq8* and can rescue the growth of *Δcoq8* yeast strain [18,19] by contributing to the stabilization of the CoQ biosynthesis complex [20]. ADCK3 has an unorthodox kinase function by harboring an ATPase activity [21]. Mutations in *ADCK3* cause autosomal recessive cerebellar ataxia with CoQ deficiency [20,22,23,24,25,26,27,28] whereas mutations in *ADCK4* promote CoQ-responsive, steroid-resistant nephrotic syndrome due to CoQ deficiency [29]. Silencing of *ADCK1* affects epithelial cell migration [30] while *ADCK2* silencing reduces the viability of both glioblastoma-derived cancer cells [31] and estrogen receptor-positive breast tumors [32]. The depletion in *ADCK2* also significantly decreases the effect of tumor necrosis factor α (TNFα) on hypoxia-inducible factor-1 (HIF-1α) stability in osteosarcoma and prostate cancer cell lines [33]. However, no information is available about the role of ADCK2 in CoQ biosynthesis and, so far, there is no disease associated with this gene [15,34,35].

Here, we report that *Adck2* haploinsufficiency in mice caused mitochondrial dysfunction that affected mainly the skeletal muscle with evidence of liver steatosis without cognitive deficits or impairment in brain function. Mitochondrial myopathy associated with CoQ deficiency in skeletal muscle was observed along with marked perturbation in whole animal mitochondrial β-oxidation. This phenotype was reminiscent of that seen in a male patient with *ADCK2* haploinsufficiency that was partially rescued by CoQ supplementation. Our results showed that *ADCK2* exerts a unique role in lipid homeostasis through control of the mitochondrial CoQ pool in muscle and organismal FA oxidation.

## 2. Materials and Methods

### 2.1. Study Approval

We obtained blood, muscle samples, and pedigrees following informed consent from individuals of the family of the patient with severe myopathy. The Ethical Committee of the Carl Gustav Carus University, Dresden, approved human subject research. A reference pathologist evaluated muscle biopsies. The Ethical Committee for Animal Experimentation of the University Pablo de Olavide approved the mouse studies on 27 May 2013, according to the European Union Directive of 22 September 2010 (2010/63/UE) and with the Spanish Royal Decree of 1 February 2013 (53/2013). All efforts were made to minimize the number of animals used and their suffering.

### 2.2. Mouse Model

The *Adck2* knockout mouse model was produced at the University of Michigan Transgenic Animal Model Core, Biomedical Research Core Facilities (Ann Arbor, MI, USA). All experiments were carried out in male mice from 6–12 months of age. Chimeras were produced by microinjecting C57BL/6-derived mutant embryonic stem (ES) cells into albino C57BL/6 host blastocysts obtained from the mating of C57BL/6-BrdCrHsd-Tyrc females with C57BL/6-BrdCrHsd-Tyrc males. Any white pups from chimera breeding have contribution from both the ES cells and the host embryos and were labeled as C57BL/6-BrdCrHsd. The ES cell clones Adck2 ACB, Adck2 AB3, Adck2 AG2 were obtained from Knockout Mouse Project (KOMP) repository, Mouse Biology Program, University of California (www.komp.org). These cells were heterozygous for the *Adck2* deletion, and the host blastocysts were wild type (WT) for the introduced mutation. The breeding of chimeric males with albino C57BL/6 females produced black pups, which were derived from the ES cells. Because the ES cells were heterozygous for the mutation, half of the black pups were expected to be positive for the mutation. Mice were supplemented with 15 mg/kg/day of CoQ_10_ (Kaneka QH stabilized powder type P30) (Kaneka Pharma Europe, Brussels, Belgium) dissolved daily in the drinking water when indicated.

### 2.3. Cell Strains and Culture

Dermal fibroblasts from the study participant (subject II-3) and his sister (subject II-2), primary human fibroblasts MRC-5 (CCL-171, American Type Culture Collection (ATCC Manassas, VA, USA), and neonatal human dermal fibroblasts (HDFs) (PCS-210-010 and PCS-210-012, ATCC) were plated in separate six-well plates (40,000 cells/well) and cultured using Dulbecco’s modified Eagle’s medium (DMEM) with 20% fetal calf serum (FCS). Mice embryonic fibroblast (MEF) preparations from WT, *Adck2*+/− and *Adck2*−/− mice were obtained from fetuses at day 9 postcoitum as described elsewhere [36].

### 2.4. Targeted Gene Sequencing 

Total DNA preparation was carried out following standard procedures. The mitochondrial and the exons and intron–exon boundaries of *ETFA, ETFB, ETFDH, ADCK1*, *ADCK3, ADCK4*, *ADCK5*, decaprenyl-diphosphate synthase subunit 1 (*PDSS1*), and subunit 2 (*PDSS2)*, *COQ2*, *COQ3*, *COQ4*, *COQ5*, *COQ6*, *COQ7*, *COQ8A*, *COQ8B*, *COQ9*, *COQ10A*, *COQ10B,* and *PPTC7* were analyzed by Sanger sequencing. For quantification of the *ADCK2* and *Adck2* transcripts, RNA was extracted with easy-BLUE Total RNA extraction kit (iNtRON Biotechnology) according to manufacturer’s instructions. One microgram of total RNA was used to obtain cDNA of human *ADCK2* gene with the iScript cDNA Synthesis Kit (Bio-Rad) following the manufacturer’s instructions. For sequencing of *ADCK2* mRNA from the index patient and other family members, RNA was isolated from cultured fibroblasts (RNeasy Plus Mini Kit-Qiagen) or from whole blood (PAXgene Blood RNA kit, PreAnalytiX), and reverse transcription was performed using the Quantitect Reverse Transcription kit (Qiagen). The entire *ADCK2* mRNA was amplified using four primer pairs with one primer from each pair spanning adjacent exons [37], and the amplicon was sequenced in the forward and reverse direction.

### 2.5. Measurement of Plasma Acyl-Carnitine and Urinary Organic Acids

Analysis of plasma acyl-carnitines in the index patient and other family members was performed on dry blood spots (Screening Labor, Hannover, Germany). For the animal studies, analysis of acyl-carnitines in plasma was performed by high-performance liquid chromatography, electrospray ionization, and tandem mass spectrometry in the underivatized form using a commercial kit of deuterated acyl-carnitines (Perkin Elmer, Barcelona, Spain). The trimethylsilylated derivatives of organic acids were determined in mouse urine by gas chromatography/mass spectrometry (5975C Agilent Technologies, Madrid, Spain). The signals (*m/z*) of the specific ions for every compound were corrected by the amount of the internal standard (undecanodioic; ion 345). For all urine samples, the same amount of creatinine was loaded onto the gas chromatograph/mass spectrometer. Plasma lactate levels were measured with a lactate oxidase–peroxidase (colorimetric) assay kit (Spinreact, Barcelona, Spain) as indicated by the manufacturer.

### 2.6. Yeast Functional Complementation

Yeast *YPL109c* mutants were transformed with the pYES2 vector containing the different versions of human *ADCK2*, and either the WT, site-directed mutant yeast *YPL109c*, or the empty vector for complementation experiments. Growth rate in non-fermentable carbon source (YPG medium) was determined as a marker of functional complementation.

### 2.7. Physical and Behavioral Tests

The rotarod performance test was performed to assess motor activity and coordination in mice. Animals were placed on an accelerating rotating rod (45 rpm max), and the time it took the mice to fall off of the rod was recorded. In the treadmill exhaustion test, the mice were forced to run on a treadmill (Treadmill Columbus 1055M-E50; Cibertec SA, Madrid, Spain) at 8% inclination until exhaustion, starting at 10 m/min and increasing the speed by 5 m/min every 5 min until it reached 25 m/min. Exhaustion was achieved when animals who quit running refused to move back to the treadmill 5 sec after receiving an electric foot shock [38]. Muscle force in mice was measured in vivo using a Grip Strength Columbus apparatus (Cibertec SA) as indicated [39]. Behavior was analyzed by passive avoidance to test associative memory and other tasks such as hot-plate sensitivity, exploratory locomotor activity by the open field actimeter [40]. Associative short- and long-term memories were determined by novel object recognition (NOR) task as described [41]. (*n* = 10 per group).

### 2.8. CoQ Determination and Biosynthesis

CoQ concentrations and biosynthesis rate were determined as described by Rodríguez-Aguilera et al. [42]. Moreover, CoQ biosynthesis rate was also calculated upon cell incubation with (^3^H)–mevalonate, an approach that allowed the measurement of cholesterol biosynthesis as well [43]. Specific activities were expressed as disintegrations per minute (DPM) min^−1^·mg protein^−1^. Stable expression of *ADCK2* in human fibroblasts was achieved using lentiviral particles. The wild-type allele of human *ADCK2* was cloned in using the lentivirus transfer vector containing chimeric Rous sarcoma virus (pRRL), with the promoter sequence required for *ADCK2* expression EF1a, and the promoter of phosphoglycerate kinase (PGK) required for the green fluorescent protein (GFP) expression (pRRL.sin.EF1a-PGK-GFP) plasmid to obtain the production of lentiviral particles (II-3-pRRL-*ADCK2*). Cell type 293T (5 × 10^6^ cells per plate) were transfected with the II-3-pRRL-*ADCK2* plasmid, along with the plasmid required to the lentivirus packaging (psPAX2) and the required to produce the virus capsid (pMD2.G). After 16 h of incubation, the culture medium was replaced with fresh medium, which was collected 48 h later and subjected to filtration and centrifugation (26,000 rpm, 2 h, 4 °C) in order to recover lentiviral particles. Human fibroblasts were seeded at 50,000 cells per well and expanded until reaching ~90% confluency, after which the medium was changed and lentiviral particles (400 µL per well) were added. Twenty-four hours later, more than 98% GFP-positive cells (a direct measure of transfection) were collected by cell sorting.

### 2.9. Mitochondrial Biochemistry 

Activities of NADH: coenzyme Q_1_ oxidoreductase (Complex I), succinate dehydrogenase (Complex II), ubiquinol: cytochrome c oxidoreductase (Complex III), NADH: cytochrome c reductase (Complex I–III), succinate: cytochrome c reductase (Complex II–III), cytochrome c oxidase (Complex IV), and citrate synthase (CS) were determined in muscle biopsy from patients, in mouse skeletal muscle, and in fibroblast lysates by spectrophotometric assays as described [44]. Oxygen consumption rate (OCR) based on glucose as energy substrate was determined with an XFe24 Extracellular Flux Analyzer (Seahorse Bioscience), as specified by the manufacturer. FA oxidation was determined by palmitate-dependent OCR in permeabilized cells as described [45]. MEFs or fibroblasts were seeded in XFe24 cell culture microplates at the density of 7.5 × 10^3^ cells per well. Data were normalized to the number of cells in each well counted at the end of the experiment. In order to perform immunostaining, cells were plated at a density of 10 × 10 cells on coverslips and were immunostained with antibodies against GFP and TOM20 as indicated [44]. Western blots were carried out as described [5].

### 2.10. Microarray Analysis

RNA was extracted from liver and skeletal muscles of six-month-old *Adck2*+/− (*n* = 5) and WT (*n* = 5) mice. Probe-target hybridization with GeneChip Mouse Genome Plus 2.0 (Affymetrix) was performed as we previously described [46]. Statistical analyses were performed comparing each signal of *Adck2*+/− with the corresponding signal of WT. Data have been deposited with the National Center for Biotechnology Information, Gene Expression Omnibus database repository (NCBI-GEO) [47] with accession number GSE104996. Microarrays results were validated by quantitative RT-PCR analysis of 10 significant genes (Table S7).

### 2.11. Untargeted Metabolomics Assay

Metabolomics analysis on mouse liver, skeletal muscle, and plasma extracts was performed by the University of California Davis, West Coast Metabolomics Center (Davis, CA, USA) according to established procedures [46]. Twelve-month-old *Adck2+/−* and WT mice were used (*n* = 6).

### 2.12. Metabolic Cages 

Mouse metabolic rate was assessed in vivo by indirect calorimetry in open-circuit oxymax chambers using the Comprehensive Lab Animal Monitoring System (CLAMS; Columbus Instruments, Columbus, OH, USA) as previously described [48]. 

### 2.13. Quantitative Real-Time PCR

Quantitative real-time PCR was performed to measure the expression of select target genes [49]. The primers were designed with the Beacon Designer software, and the primer pair sequences used in this study were as followed: human *ADCK2*, forward primer 5’-CAGGAAGAACACCATCAC-3′ and reverse primer 5’-TGAGTCATCAGCAACTTAA-3’; mouse *Adck2*, forward primer 5’-TAAGTCAGATCACCTCAT-3’ and reverse primer 5’-CAATCTTCATCAGCAGTA-3’; human glyceraldehyde 3-phosphate dehydrogenase (*GAPDH*), forward primer 5’-TGCACACCACCAACTGCTTAGC-3′ and reverse primer 5′-GGCATGGACTGTGGTCATGAG-3′; and mouse *Gapdh*, forward primer 5′-TGACGTGCCGCCTGGAGAAA-3′ and reverse primer 5’-AGTGTAGCCCAAGATGCCCTTCAG-3′.

The following primer pairs were used (F, forward orientation; R, reverse orientation; sequence 5′ to 3’) to validate microarray results (Table S7): apolipoprotein (apo-) A-II, ApoA2-F (TGGTCGCACTGCTGGTAAC), ApoA2-R (TTTGCCATATTCAGTCATGCTCT), homeobox protein, Cdx4-F (TGACATGACCTCCCCAGTTTT), Cdx4-R (GCCGGAGTCAAGAGAAACCA), Cathepsin H, Ctsh-F (ACCGTGAACGCCATAGAAAAG), Ctsh-R (TGAGCAATTCTGAGGCTCTGA), glutathione S-transferase P 1, Gstp1-F (ATGCCACCATACACCATTGTC), Gstp1-R (GGGAGCTGCCCATACAGAC), melanoma inhibitory activity protein 2, Mia2-F (GTGTCTGGAGGGTACAAAGTTG), Mia2-R (TCGGGTCCTGTGTAATCTCTC), NADH:ubiquinone oxidoreductase subunit B2, Ndufb2-F (CCCCGGTACAGGGAGTTTC), Ndufb2-R (GCCAAAATCGCCAAAGAATCCA), pleckstrin homology-like domain family A member 2, Phlda2-F (CTCCGACGAGATCCTTTGCG), Phla2-R (ACACGTACTTAGAGGTGTGCTC), solute carrier family 2 member 2, Slc2a2-F (TCAGAAGACAAGATCACCGGA) Slc2a2-R (GCTGGTGTGACTGTAAGTGGG), translocase of outer mitochondrial membrane 7, Tomm7-F (ATCCGCTGGGGCTTTATTCC), Tomm7-R (CGACGGTTCAGGCATTCCA), transferrin, Trf-F (GCTGTCCCTGACAAAACGGT), Trf-R (CGGAAGGACGGTCTTCATGTG).

### 2.14. ADCK2 Silencing by siRNA

Small inhibitory RNAs (siRNA) against human *ADCK2* (Hs_ADCK2_6) were purchased from Qiagen (Hilden, Germany) and had the following sequences: sense strand 5’-GAUUGACCUGCGUUACGAA-3′, antisense strand 5′-UUCGUAACGCAGGUCAAUC-3’, and the target sequence 5’-CAGATTGACCTGTACGAA-3’. Cells were seeded in a 60-mm dish at 3.5 × 10^5^ cells/plate 24 h prior to transfection and were then transfected with 0.4 nmol of siRNA using oligofectamine (Invitrogen) as transfection reagent. Cells were harvested and analyzed 72 h after transfection. The knockdown of *ADCK2* was confirmed by quantitative real-time PCR.

### 2.15. Antibodies

Primary antibodies were used against ADCK2 (custom antibody from Biomedal, 1:1000), ADCK2 (Byorbit, orb100461, 1:500), translocase of outer mitochondrial membrane 20 (Tomm20) (Santa Cruz, sc-11415, 1:10000), mitochondrial dynamin like GTPase (Opa1) (BD Biosciences, 612606, 1:1000), ornithine aminotransferase (OAT) (Acris Antibodies, AMO9362PU-S, 1:1000), Calnexin (Stressgen, ADI-SPA-860, 1:1500), mitofusin 2 (Mfn2) (Abcam, ab50843, 1:1000) and lactate dehydrogenase (LDH) (Rockland, 100-1173, 1:2000).

### 2.16. Statistics

Statistical comparisons between animals of both genotypes were assessed using paired Student’s *t*-test. Analyses were performed using Excel 2010 (Microsoft Corp., Redmond, WA, USA). LogRank statistical analyses were performed using Sigmastat 3.5 (Systat Software Inc, San Jose, CA, USA). For behavior tests, comparisons between groups were made using a Student’s *t*-test, and the differences between sessions were analyzed with repeated measures ANOVA using SPSS 18 software (Madrid, Spain). Results are expressed as the mean ± SD and differences were considered significant at *p* values ≤ 0.05.

## 3. Results

### 3.1. Haploinsufficiency of Human ADCK2 Caused a Defect in Lipid Catabolism and CoQ_10_-Deficient Myopathy

**Figure 1:**
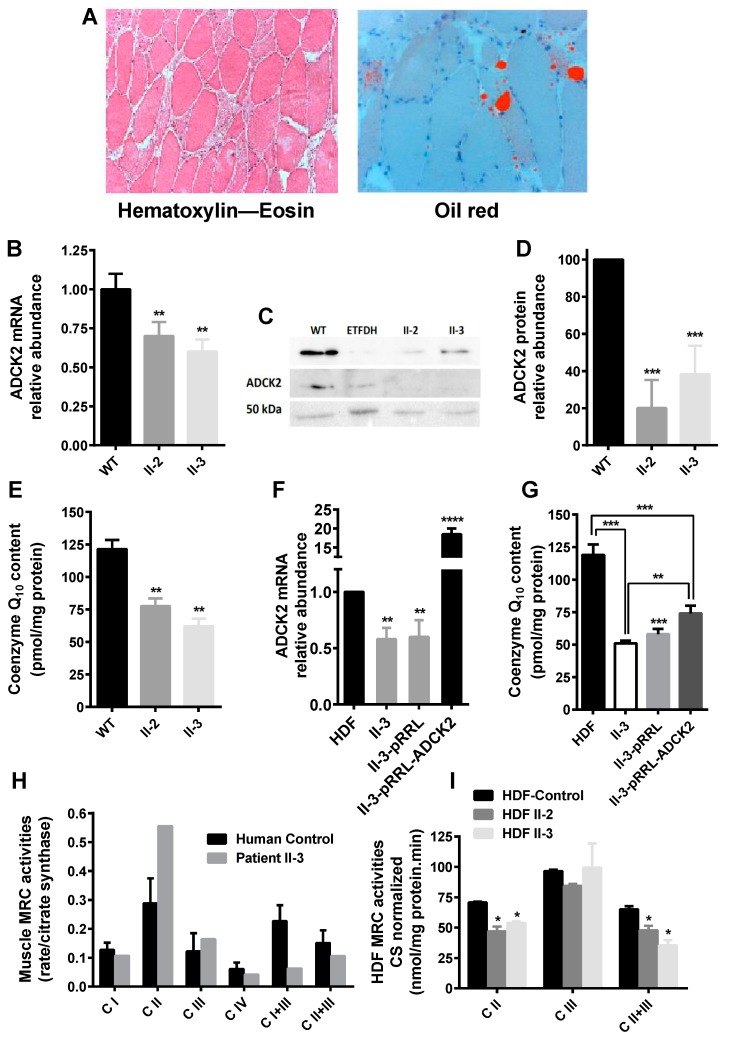
Laboratory findings in the index patient. (**A**) Histological findings in muscle of patient II-3: (left picture) some fiber atrophy and fibers containing numerous vacuoles were apparent with the haematoxylin–eosin stain (×10); (right picture) Gomori trichrome stain showing lipid droplets in some muscle fibers (×20). (**B**) Measurement of the amount of aarF domain-containing mitochondrial protein kinase 2 (*ADCK2)* mRNA in cultured fibroblasts from II-3 and II-2 relative to controls. (**C**) Western blotting of ADCK2 in cultured fibroblasts from II-3 and II-2. (**D**) Quantification of the protein expression levels measured in (C). (**E**) Coenzyme Q (CoQ) content in dermal fibroblasts from patients II-3 and II-2 (*n* = 5). Expression level of *ADCK2* mRNA (**F**) and CoQ content (**G**) in patient fibroblasts transformed with the *ADCK2* wild type (WT) allele (*n* = 5). (**H**) Mitochondrial respiratory chain (MRC) activities in muscle biopsy of patient II-3 normalized to citrate synthase (CS). The complexes III and IV values shown are ×10^−2^. (**I**) MRC activities in human dermal fibroblasts (HDF) from control, patient II-3, and his sister II-2. * *p* < 0.05, ** *p* ≤ 0.01, *** *p* ≤ 0.005 vs. control/WT, **** *p* < 0.001 vs. non-transformed/empty vector; *n* = 10. Data were analyzed using Student’s *t* test.

The index patient, a 50-year-old man, (subject II-3 (Figure S1A)), developed a slow, progressive proximal muscle weakness with permanent myalgia both at rest and during muscular exertion, resulting in permanent disability. In the end, the patient required a wheelchair. Muscle MRI revealed severe fatty degeneration of the shoulder girdle, deltoid, biceps, hamstring, and calf muscles, whilst the triceps was well preserved (Figure S1B). The patient developed a lipid storage myopathy and liver dysfunction. Histological analysis of skeletal muscle demonstrated mitochondrial myopathy associated with lipid droplets (Figure 1A). The patient had normal cognitive function and did not show ataxia. Analysis of the mitochondrial genome was negative. Sequencing of a set of genes potentially involved with primary or secondary CoQ deficiency showed the heterozygous NM_052853.3:c.997C>T; p. (Arg333*) mutation in exon 2 of *ADCK2* in both the forward and reverse sequences, which was absent in 50 subjects from the same ancestry. The variant is absent among the ~13,005 alleles listed on the National Heart, Lung, and Blood Institute (NHLBI) exome sequencing project exome variant server [50] and is not listed on the 1000 genomes project database, and has a minor allelic frequency (MAF) of 2:251,006 according to the Genome Aggregation Database of Broad Institute of Harvard University and Massachusetts Institute of Technology [51]. Although his mother and sister harbor the same mutation, they do not currently have any neuromuscular symptoms (Figure S1C). The mutation produced a termination codon that led to a significant decrease in ADCK2 mRNA and protein levels in dermal fibroblasts of the patient (II-3) and his sister (II-2) (Figure 1B–D), both showing a significant reduction in CoQ_10_ levels (Figure 1E). Transfection of these dermal fibroblasts with the WT *ADCK2* allele rescued the ADCK2 mRNA levels (Figure 1F) and CoQ_10_ content (Figure 1G). The reduction in complexes I+III and II+III activities of muscle (Figure 1H) and dermal fibroblasts (Figure 1I) from II-3 and II-2 was indicative of a CoQ deficiency. 

We observed a moderate elevation in plasma levels of saturated short- and medium-chain length acyl-carnitine species (Table S1, Column 1), consistent with multiple acyl-CoA dehydrogenase deficiencies. Patient II-3 was treated with 75 mg CoQ_10_ per day (Figure S1B–E), showing an improvement in the plasma lactate and myoglobin levels (Figure S1). One month later, the patient stopped taking CoQ_10_, but after three weeks his pain worsened, his muscle strength decreased, and he lost the ability to lift his arms. Thus, it would appear that the symptoms were partially dependent on CoQ_10_. A complete description of the pathological phenotype of the patient is presented in the Supplementary Materials.

### 3.2. A Heterozygous Adck2 Knockout Mouse Model Recapitulated the Phenotype Observed in Patient II-3

**Figure 2:**
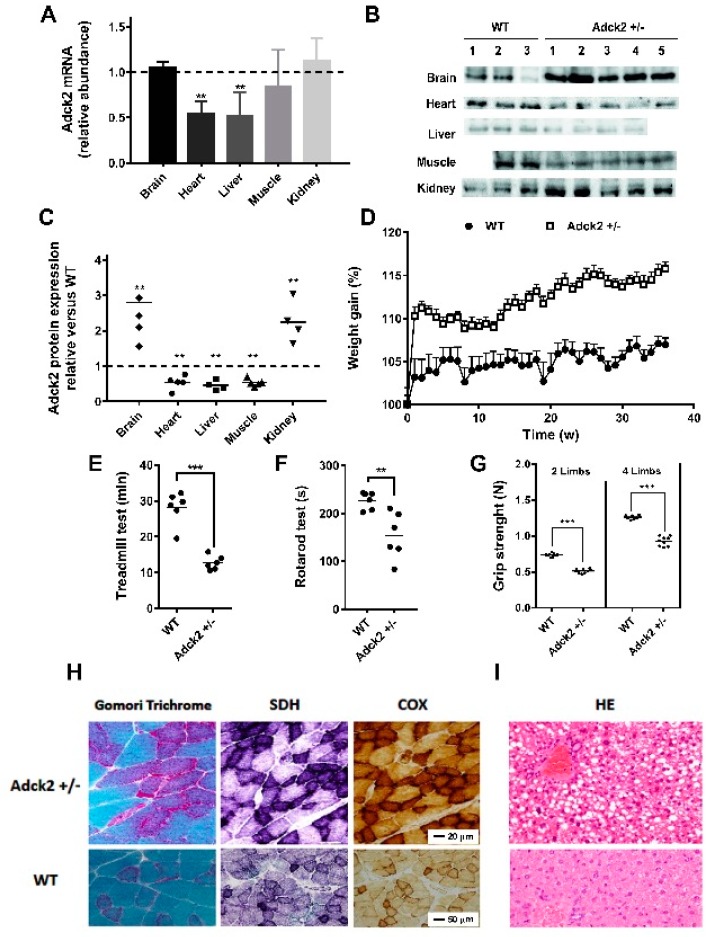
Characteristics of the *Adck2+/−* mouse model. (**A**) Relative abundance of *Adck2* mRNA levels in different tissues of *Adck2*+/− mice after normalization to WT controls, depicted as a dotted line. Bars represent mean + SD; ** *p* < 0.01 (*n* = 5 per group). (**B**) Western blot of ADCK2 protein in different mouse tissues. (**C**) Densitometry of ADCK2 protein levels from panel (B) (*n* = 4–5). (**D**) Weight gain trajectories of WT and *Adck2*+/− male mice on standard diet (*n* = 15). (**E**) Physical activity as determined by the running time on a treadmill until exhaustion. (**F**) The latency period before falling off an accelerating rotarod. (**G**) Grip strength capacity. ** *p* < 0.01 vs. WT; *** *p* < 0.005 vs. WT; *n* = 6. Data were analyzed by one-way ANOVA (D) and Student’s t test (panels A, B and E–G). (**H**) Skeletal muscle of *Adck2*+/− mice stained with Gomori trichrome, succinate dehydrogenase (SDH), and cytochrome c oxidase (COX) were compared to WT; 60× magnification. (**I**) Light microscopy of mouse *Adck2*+/− liver showing extensive steatosis compared to WT liver after hematoxylin and eosin (HE) staining; 40× magnification.

A heterozygous (*Adck2*+/−) knockout mouse model was developed based on the high homology and sequence conservation of this gene across different species. The *Adck2* mRNA expression was significantly lower in liver and heart, with a trend toward reduced expression in skeletal muscle, but without change in brain and kidney (Figure 2A). Adck2 protein was also decreased in heart, liver, and skeletal muscle, but was increased in both brain and kidney as compared to wild type (WT) littermates (Figure 2B,C). Heterozygote pups weighed significantly less at weaning than WT (*Adck2*+/−: 15.84 ± 0.64 g (*n* = 10), WT: 18.23 ± 0.35 g *(n* = 13), *p* ≤ 0.003) and showed a trend toward greater weight gain with age (Figure 2D). No significant difference in body weight was noted for most of the time points analyzed (Figure S2A) despite visceral fat accumulation (Figure S2B). Male *Adck2*+/− mice (12-month-old) showed significantly reduced overall endurance, as evidenced by shorter running distance on a treadmill due to exhaustion (Figure 2E) and poorer motor coordination on a rotarod (Figure 2F). A decline in two and four limb grip strength tests was also observed in the mutant mice (Figure 2G). Thus, *Adck2* haploinsufficiency caused locomotor dysfunction in mice.

Pathological laboratory findings in the *Adck2*+/− mice included significant increases in plasma lactate (in mmol/L, 7.1 ± 0.2 for *Adck2*+/− vs. 3.4 ± 0.08 for WT; *p* < 0.05, *n* = 7) and accumulation of organic acids in the urine (Table S2). A non-significant increase in transaminases was observed in the plasma of six-month-old mice: glutamate-pyruvate transaminase/alanine transaminase (GPT/ALT) (in mg/dL, 52.2 ± 6.1 in WT and 56.3 ± 5.5 in *Adck2*+/−; *p* = 0.937, *n* = 5) and glutamic oxaloacetic transaminase/aspartate transaminase (GOT/AST) (in mg/dL, 464.8 ± 120 in WT and 660.7 ± 122 in *Adck2*+/−; *p* = 0.426, *n* = 5).

Gomori trichrome stain of gastrocnemius muscle from *Adck2*+/− mice showed a mild variation in fiber size, and some fibers might have been classified as ragged red fibers (Figure 2H). Both the succinate dehydrogenase (SDH) and cytochrome *c* oxidase (COX) staining were increased in *Adck2*+/− muscle fibers compared with WT (Figure 2H), consistent with mitochondrial proliferation. Hematoxylin and eosin (HE) analysis of liver from *Adck2*+/− mice showed an accumulation of large lipid droplets in periportal and perivenular hepatocytes compared to WT littermates (Figure 2I).

We performed memory and neurobehavioral assessments in 12-month-old *Adck2*+/− mice. Using the hot-plate test to measure the pain response, no differences were observed in both the latency period and the behavioral reaction to pain caused by heat (Figure S3A), indicating unaltered nociceptive information and emotional response in the *Adck2*+/− mice. The fear-conditioning test indicated that both groups of mice entered the dark compartment with similar latencies, and in the retention test WT and *Adck2*+/− mice displayed the same responses (Figure S3A). The novel object recognition (NOR) task evaluated short- and long-term memories. The total number of object contacts made by *Adck2*+/− mice was somewhat higher during the training session and after a latency period (Figure S3B). A tendency toward more extensive exploratory behavior of *Adck2*+/− mice in the open field test was recorded when compared to WT controls (Figure S3B). Similarly, there were no differences in the Recognition Index in any session in both groups of mice (Figure S3C). Taken together, these results demonstrated the absence of overt central nervous system pathology in *Adck2*+/− mice.

### 3.3. Adck2 Deficiency Caused CoQ Deficiency in MEFs and Skeletal Muscle

**Figure 3:**
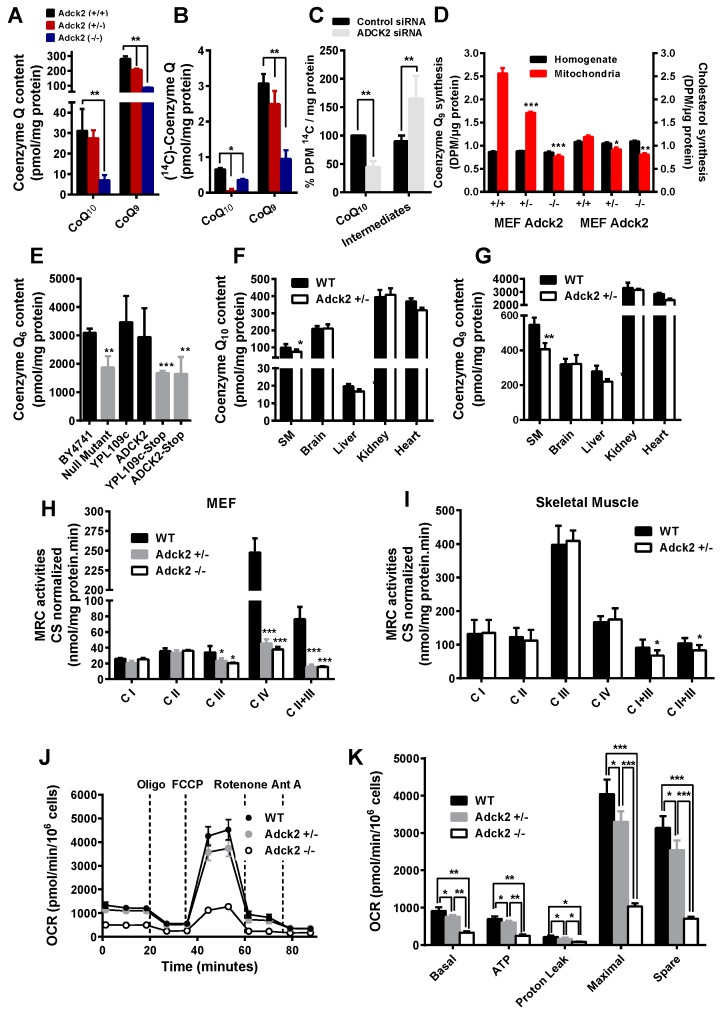
Coenzyme Q content and MRC activities in cells and tissues. CoQ_9_ and CoQ_10_ content (**A**) and biosynthesis (**B**) in mice embryonic fibroblasts (MEFs) isolated from Adck2−/−, *Adck2+/−* and WT mice (*n* = 5). (**C**) Effect of siRNA silencing of *ADCK2* on the rate of CoQ_10_ biosynthesis, measured by incorporation of ^14^C-*p*-hydroxybenzoate (^14^C-*p*-HB) intermediate, in MRC5 human fibroblasts (*n* = 4). (**D**) Levels of CoQ (first three sets of bars) and cholesterol (second three sets of bars) produced in the presence of ^3^H-mevalonate in total homogenates and mitochondrial fractions from MEFs. (**E**) CoQ_6_ content in BY4741 WT and *ΔYPL109c* (null mutant) yeast strains after transformation of *ΔYPL109c* with *YPL109c* WT or human *ADCK2*. Also, *ΔYPL109c* was transformed with mutant alleles of either *YPL109c* (*YPL109c*-stop) or *ADCK2* (ADCK2-stop) (*n* = 7). The content in CoQ_10_ (**F**) and CoQ_9_ (**G**) was determined in various tissues and organs of *Adck2*+/− and WT mice (*n* = 5). MRC activities normalized to citrate synthase in MEFs (**H**) and skeletal muscle (**I**) of both WT and *Adck2*+/− mice. (**J**) and (**K**) Oxygen consumption rate (OCR) in WT, *Adck2+/−* and *Adck2*−/− MEFs. Bars are mean + SD. Data was analyzed by using repeated-measures ANOVA test. (*n* = 5); * *p* < 0.05, ** *p* < 0.01, and *** *p* < 0.005 vs. WT.

Similar to the patient’s fibroblasts and muscle, cultures of mouse embryonic fibroblasts (MEFs) isolated from nine day-old *Adck2*−/− and *Adck2*+/− embryos exhibited a significant reduction in both CoQ_9_ and CoQ_10_ levels (Figure 3A) and in their biosynthetic rates as determined by the incorporation of ^14^C-*p*-hydroxybenzoate (*p*-HB) (Figure 3B). *ADCK2* knockdown by siRNA interference induced a significant decrease in CoQ biosynthesis rate in human MRC5 cells (Figure 3C). The rate of incorporation of the isoprenoid moiety in CoQ was measured by incubating MEFs with ^3^H-mevalonate in the presence of an excess of non-labeled *p*-HB. Regardless of the genotype, the levels of radiolabeled CoQ and cholesterol were unchanged in total MEF homogenates; however, the mitochondrial fraction of *Adck2*-deficient MEFs had significantly lower levels of both molecules, which indicated a defect in intracellular trafficking of isoprenoid and cholesterol from the cytoplasm, where they are synthesized de novo, in mitochondria (Figure 3D).

Complementation assays in *Saccharomyces cerevisiae* null strains have been employed to validate the role of *ADCK2* in CoQ biosynthesis. The deletion of *YPL109c*, the *S. cerevisiae* homolog of human *ADCK2*, caused a 40% decrease in the production of CoQ_6_, the specific isoform in yeast (Figure 3E). Transformation of the *ΔYPL109c* yeast strain with wild type *YPL109c* or human *ADCK2* construct rescued CoQ_6_ biosynthesis, whereas a mutant allele with a stop codon or the yeast gene with an equivalent stop codon failed to complement the mutant strain (Figure 3E). 

CoQ_9_ and CoQ_10_ concentrations were also significantly lower in skeletal muscle of *Adck2*+/− mice compared to WT controls, with a downward trend in the heart and liver, but not in the brain or kidney of *Adck2*+/− animals (Figure 3F,G). Collectively, these results supported the role of *ADCK2*-encoded protein in the biosynthesis of mitochondrial CoQ in both mammal and yeast cells, mainly affecting skeletal muscle of *Adck2+/−* mice.

### 3.4. Adck2 Haploinsufficiency Caused Mitochondrial Dysfunction

Immunoblotting in various subcellular fractions of HEK293 cells showed a distribution of ADCK2 similar to the mitochondrial TOM20 and Mfn2 (Figure S4A,B). The presence of ADCK2 was not detected in the endoplasmic reticulum and cytosolic fraction. Both the translocase of outer mitochondrial membrane 20 (Tomm20) and calnexin, a protein present in the mitochondrial-enriched fraction and endoplasmic reticulum, were susceptible to proteinase K treatment, whereas the inner mitochondrial membrane protein OPA1, the matrix protein ornithine aminotransferase, and ADCK2 were resistant to this treatment. However, mitochondrial membrane disruption with the detergent Triton X-100 prior to proteinase K treatment caused the digestion of ADCK2, consistent with its presence in the mitochondrial matrix or bound to the inner mitochondrial membrane (Figure S4C).

The urinary excretion of organic acids, such as lactate, was significantly increased in *Adck2*+/− mice (Table S2), as was the blood lactate level (in mmol/L*,* 7.1 ± 0.2 for *Adck2*+/− versus 3.4 ± 0.08 for WT; *p* < 0.05, *n* = 7), indicating possible defects in mitochondrial oxidative metabolism. MEFs isolated from mutant mice embryos showed a significant decrease in mitochondrial complexes III, II+III, and IV activities (Figure 3H). Mitochondrial complex I+III and II+III activities were significantly decreased in *Adck2*+/− skeletal muscle, but the activities of the individual complexes were normal (Figure 3I). In contrast, complex I, II, and I+III activities were significantly higher in kidney mitochondria of *Adck2*+/− mice, whilst those of complex III, IV, and II+III were not affected (Figure S5A). The mitochondrial respiratory complexes were unaffected in the brain and liver of *Adck2*+/− mice (Figure S5B,C). The analysis of oxygen consumption rate (OCR) using the Seahorse XF Extracellular Flux Analyzer showed a significant difference in basal and maximum respiration in WT compared to *Adck2+/−* and *Adck2−/−* MEFs when glucose was used as substrate (Figure 3J,K). Taken together, these data suggested that ADCK2 deficiency impaired the normal MRC function in MEFs and skeletal muscle mitochondria ultimately leading to mitochondrial dysfunction.

### 3.5. Adck2 Haploinsufficiency Decreased Mitochondrial Fatty Acid β-Oxidation Required to Promote Cellular Growth 

**Figure 4:**
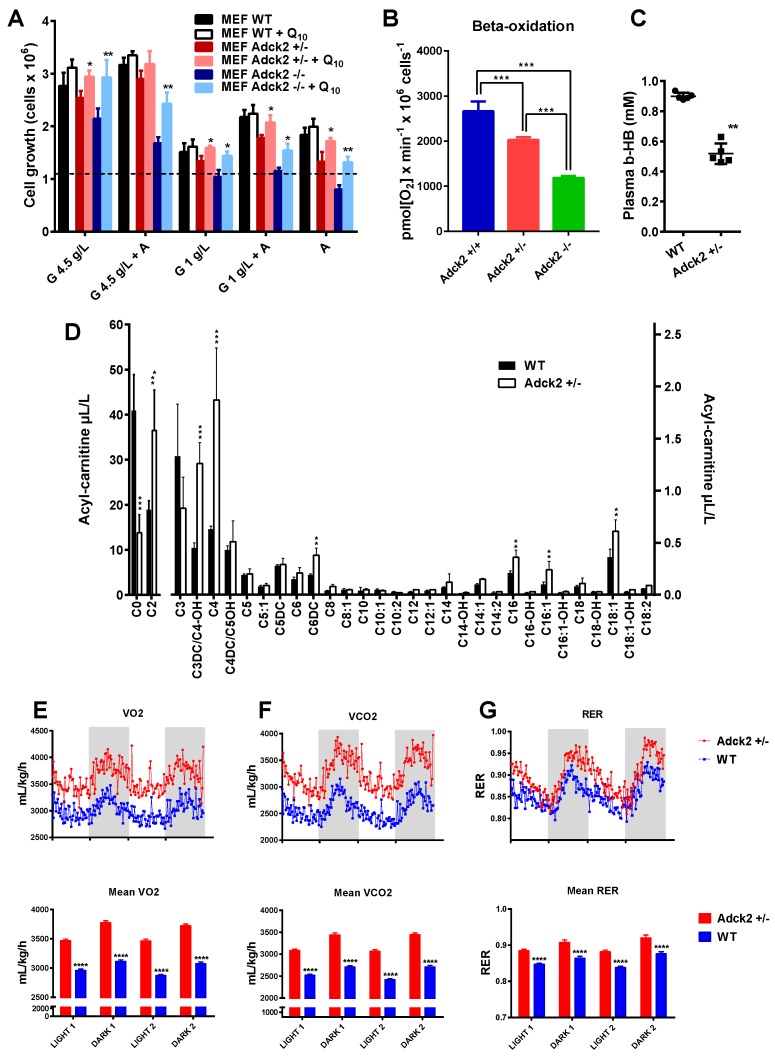
Metabolic changes associated with *Adck2* insufficiency. (**A**) Effects of glucose and FA as carbon source on cell proliferation. MEFs were grown for 72 h in media containing either high glucose (4.5g/L), low glucose (1g/L), or 1 mg/mL fatty acids (‘A’, AlbuMax©) supplemented or not with 30 nM CoQ_10_. * *p* ≤ 0.05, ** *p* ≤ 0.01 vs. non-supplemented; (*n* = 5). (**B**) Oxygen consumption rate of MEFs using palmitate as substrate. (**C**) Circulating concentrations of β-hydroxybutyrate in *Adck2*+/− and WT mice. ** *p* ≤ 0.01 vs. WT. (**D**) Acyl-carnitine profile measured in plasma of WT and *Adck2*+/− mice. ** *p* ≤ 0.01, *** *p* ≤ 0.005 vs. WT. Metabolic cages analysis of O_2_ consumption (**E**), CO_2_ production (**F**), and respiratory exchange ratio (RER) (**G)** during two fed–fasted periods comparing *Adck2+/−* and WT mice (*n* = 6). **** *p* < 0.001 vs. WT. Data were analyzed using Student’s *t* test.

Here, we tested the hypothesis that ADCK2 is required to meet the energy demands for cellular growth by upregulating mitochondrial FA β-oxidation. As anticipated, WT and *Adck2*+/− MEF growth rate was reduced when maintained in low glucose medium (e.g., 1 g/L glucose) (Figure 4A). Addition of FA to medium at a low glucose concentration increased the proliferation of WT, but not mutant MEFs, whereas FA supplementation to glucose-free medium caused more than 50% growth arrest of *Adck2*+/− MEFs compared to WT cells (Figure 4A). Moreover, palmitate-dependent OCR decreased in permeabilized MEFs lacking *Adck2* compared to WT cells (Figure 4B). The results of these combined studies indicated that ADCK2 was required to meet the energy demands for cell growth via FA β-oxidation.

Defects in mitochondrial FA oxidation have been linked with lower plasma β-hydroxybutyrate levels [52]. Here, circulating concentrations of β-hydroxybutyrate (Figure 4C) and free carnitine (Figure 4D) were significantly reduced, while short- and long-chain fatty acid levels were higher in male *Adck2*+/− mice compared to WT littermates (Figure 4D). Significantly higher urinary excretion of adipic and ethylmalonic acids was found in *Adck2*+/− mice (Table S2). These phenotypic changes were reminiscent of the patient’s impairment in FA oxidation.

Next, we used the indirect respiration calorimetry system (CLAMS metabolic chamber) to determine the preferred source of energy (fat vs. carbohydrates) for WT and *Adck2*+/− mice [48]. The respiratory exchange ratio (RER) is calculated using the ratio of CO_2_ produced over O_2_ consumed; RER of 1.0 indicates the predominant use of carbohydrates for energy production while RER of 0.7 is indicative of the preferential use of lipids as fuel source. *Adck2*+/− mice showed significantly higher oxygen consumption (Figure 4E), CO_2_ production (Figure 4F), and RER (~0.9) as compared to WT mice that had a RER of ~0.85 (Figure 4G). These data were consistent with greater use of carbohydrates by the mutant mice and consumption of a mix of fat and carbohydrates by the WT.

### 3.6. Adck2 Haploinsufficiency Affected the Gene Expression Profile in Muscle and Liver

**Figure 5:**
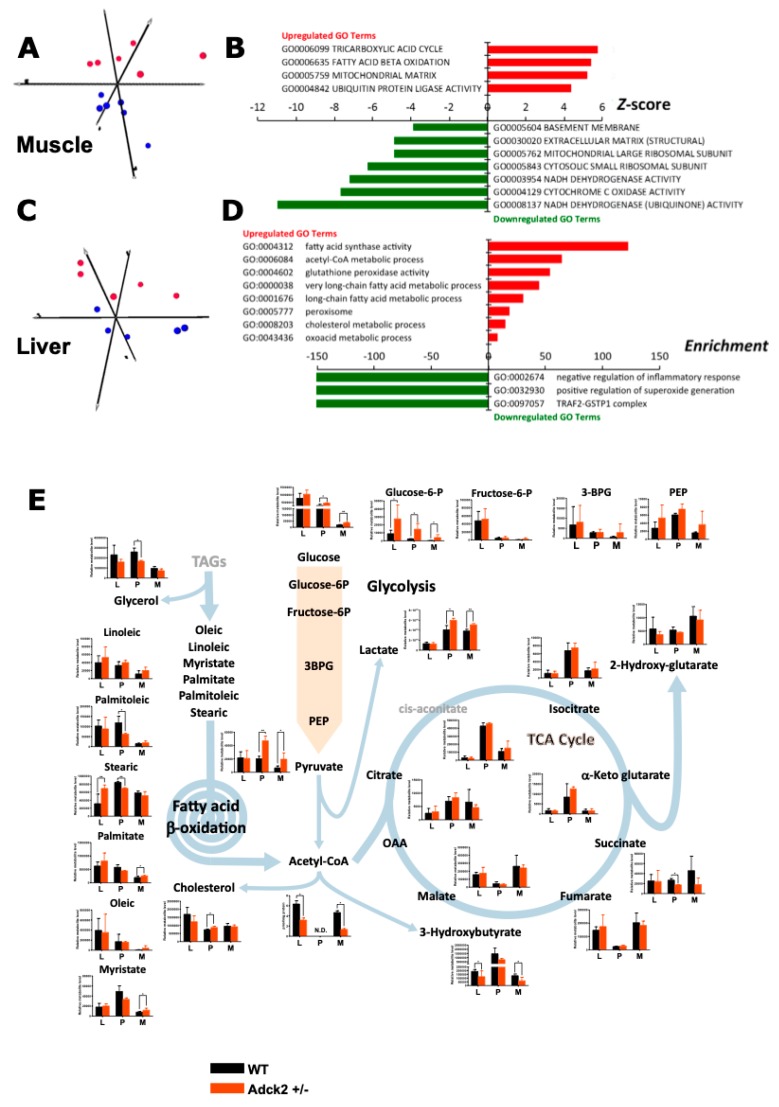
Transcriptome and metabolomics analyses in *Adck2+/−* mice. (**A**) Principal component analysis from microarray results in skeletal muscle of *Adck2+/−* (red symbols) and WT (blue symbols) mice (*n* = 6 per group). (**B**) Bars depict gene ontology (GO) pathways significantly regulated in skeletal muscle of *Adck2+/−* vs. WT animals. (**C**) Principal component analysis from microarray results in liver of *Adck2+/−* (red) and WT (blue) mice (*n* = 6 per group). (**D**) Bars depict GO pathways significantly regulated in liver of *Adck2+/−* vs. WT animals. (**E**) Metabolite profiles in skeletal muscle (M), liver (L), and plasma (P) of *Adck2+/−* and WT mice at 12 months of age are depicted. Results indicated major affected components of glycolysis and FA β-oxidation pathways, as well as the Krebs cycle (*n* = 6 per group). Acetyl-CoA was determined independently by HPLC in liver and skeletal muscle. N.D. Non determined. All data are *n* = 5–6 biological replicates per experimental group. * *p* < 0.05, ** *p* < 0.01 vs. WT. Data were analyzed using Student’s *t* test.

To further characterize the effect of *Adck2* haploinsufficiency, we performed microarray assays of RNA isolated from skeletal muscle and liver of WT and *Adck2*+/− mice. Heat map analysis showed extensive changes in the global transcript expression levels between WT and *Adck2*+/− in these tissues (Figure S6A,B). Principal component analysis based on significant Z-scores demonstrated a clear effect of *Adck2* haploinsufficiency in muscle (Figure 5A) and liver (Figure 5C). Ingenuity pathway analysis indicated a robust induction in the expression of genes related to muscle development and different myopathic conditions upon *Adck2* depletion (Figure 5B, Table S3). Major regulated genes included several biomarkers of muscle diseases, such as *Car3*, *Fbxo32,* and *Ttid*, which are overexpressed in Duchene muscle dystrophy and muscle atrophy. Additionally, *Gde1*, which is involved in skeletal muscle development, *Pdlim5*, a promoter of cardiac hypertrophy, and *Ppp1cb*, a regulator of cell division, glycogen metabolism, and muscle contractility, were overexpressed. Among the repressed genes included those implicated in muscle contractility such as *Phpt1* and *Fxyd1*. Several subunits of both cytosolic and mitochondria ribosomes (*Rps26*, *Mrpl52,* and *Rpl28*) and genes related to the control of cell proliferation, apoptosis, and mitochondrial morphology (*Romo1* and *Lgals1*) were also repressed in *Adck2*+/− muscle. Gene ontology enrichment analysis demonstrated that mitochondrial metabolism and diverse ion transport pathways were affected with concomitant repression in the activities of mitochondrial respiratory complexes, ribosomes, and protein synthesis (Table S4). Figure S6C summarizes the metabolic pathways affected by *Adck2* insufficiency in mouse skeletal muscle based on the information obtained from microarrays (Table S4).

Among the most significantly activated genes in the liver of *Adck2*+/− mice (Table S5) were four cytochrome P450s, two genes related with lipoproteins (*Vldlr*, *Srebp2*), a number of genes involved in the metabolism of cholesterol (*Mvd*), fatty acids (*Aacs*, *Acot1*, *Acot2*, *Acot4*, *Crat*, *Elovl1*, *Fasn*) and other organic acids (*Pgpep1*, *Pdk4*). Gene ontology analysis showed a general activation of FA metabolism (Figure 5D, Table S6), which could explain the liver steatosis in *Adck2*+/− mice. There was also an enrichment of genes associated with the metabolism of organic acid, purine, and cholesterol. Top down-regulated genes featured those implicated both in the repression of interleukin production and inactivation of the c-Jun N-terminal kinase pathway, consistent with activation of the hepatic inflammatory response (Figure 5D. Table S6). Expression of the DNA methyltransferase *Dnmt3b* was significantly higher and that of *Gstp1* was significantly lower in the *Adck2*+/− liver, supporting the idea of an uptick in hepatic inflammation [53]. Microarray gene expression profiles in muscle and liver were validated by quantitative real-time PCR (Table S7). 

### 3.7. Adck2 Haploinsufficiency Affected the Metabolomics Profile

To better understand the metabolic adaptation of mice to *Adck2* insufficiency, we performed untargeted metabolomics in the liver, plasma, and skeletal muscle of 12-month-old mice of both genotypes. The metabolite profile was compatible with mitochondrial dysfunction in skeletal muscle and defective mitochondrial fatty acid oxidation in the liver and muscle of *Adck2*+/− mice (Figure 5E).

Accumulation of pyruvate and lactate in plasma and skeletal muscle of the mutant mice was consistent with mitochondrial dysfunction, which decreased acetyl-CoA availability for the Krebs cycle in muscle and liver (Figure 5E). The altered production and/or clearance of these intermediates may have accounted for the significant accumulation of glucose and glucose-6-phosphate, along with a positive trend toward other glycolytic intermediates. The decrease in the Krebs cycle intermediate succinate, a mitochondrial complex II substrate and novel regulator of metabolic signaling [54], is worth of mention.

The defect in mitochondrial FA β-oxidation in *Adck2+/−* mice (Figure 4) was consistent with the significant decrease of β-hydroxybutyrate and glycerol, and a trend of cholesterol increase in plasma of *Adck2+/−* mice supported a lipid metabolism disorder. This was further evidenced by the accumulation of free FA such as linoleic, myristic, and palmitic in tissues (notably stearic acid in the liver) and their decrease in plasma. Such bioenergetics defects in *Adck2+/−* mice may have explained the in vivo metabolic cage data (Figure 4G).

### 3.8. CoQ Supplementation Partially Rescued the Adck2+/− Phenotype

**Figure 6:**
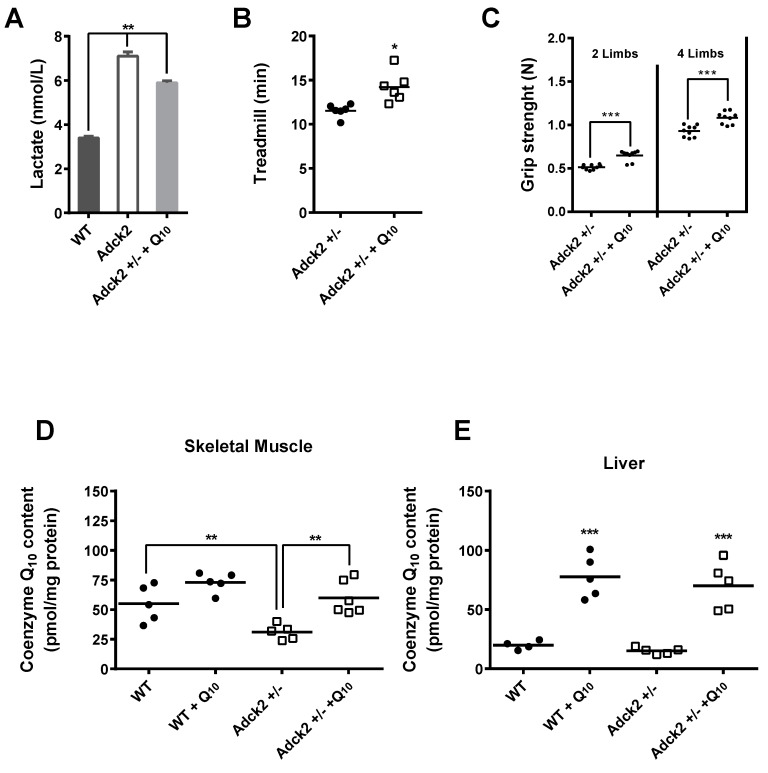
Supplementation with CoQ_10_ partially rescued the *Adck2*+/− phenotype. (**A**) Six-month-old *Adck2*+/− mice were treated or not with 15 mg/kg of CoQ daily for three months followed by the determination of plasma lactate. (**B**) Running capacity on treadmill. (**C**) Grip strength (*n* = 6). CoQ_10_ content in skeletal muscle (**D**) and liver (**E**) of both WT and *Adck2*+/− mice in the absence and presence of CoQ supplementation (*n* = 7). * *p* ≤ 0.05, ** *p* ≤ 0.01, *** *p* ≤ 0.005 vs. non-treated animals. Data were analyzed by Student’s *t* test.

The effect of CoQ_10_ supplementation in *Adck2*+/− mice was carried out to determine whether CoQ deficiency per se contributed to the overall phenotypic changes. Daily treatment of male *Adck2*+/− mice with 15 mg/kg CoQ_10_ for three months induced a significant decrease in plasma lactate levels (Figure 6A), significantly improved both the treadmill endurance exercise time (Figure 6B) and grip strength (Figure 6C). Significant incorporation of CoQ_10_ was found in skeletal muscle of *Adck2*+/−, but not WT mice (Figure 6D).

Supplementation also induced an increase of CoQ_10_ in liver of both *Adck2+/−* and WT mice (Figure 6E). Although the effect was not significant, CoQ ingestion caused a ~11% reduction in serum transaminases in *Adck2*+/− mice: GPT/ALT (in mg/dL, 50.5 ± 4.7 in CoQ-treated vs. 56.3 ± 5.5 in non-treated; *p* = 0.851, *n* = 7) and GOT/AST (in mg/dL, 578 ± 120 in CoQ-treated vs. 660.7 ± 122 in non-treated; *p* = 0.236, *n* = 7). These results demonstrated a partial role of CoQ deficiency in the pathogenesis of the *Adck2+/−* mouse myopathy and liver dysfunction. 

## 4. Discussion

In the work herein, we have demonstrated the involvement of ADCK2 in the mitochondrial import of CoQ precursors and the impact that a heterozygous nonsense mutation in *ADCK2* has on mitochondrial function and the rate of FA oxidation in skeletal muscle of a patient and in a mouse model. As the patient showed a late onset of the disease, we have studied adult animals ranging from 6 to 12 months old. A nonsense mutation in one *ADCK2* allele led to the development of severe mitochondrial myopathy in a human patient that appeared responsive to CoQ supplementation. 

The sister of this patient also harbors the *ADCK2* mutation, but she did not complain of neuromuscular symptoms at this time. Nevertheless, she displayed CoQ deficiency in cultured fibroblasts. Adult onset autosomal dominant diseases often display incomplete penetrance and variable expressivity. This is consistent with the detection of the same *ADCK2* mutation in two apparently healthy controls in the gnomAD database. Several factors may act as phenotype modifiers in dominant disorders. In the case of facioscapulohumeral dystrophy, estrogens appear to have a protective role [55], but many other genetic and environmental factors may influence the phenotype. 

There are at least two reports of patients with isolated myopathy associated with muscle CoQ deficiency and abnormal acyl-carnitine profile in the absence of brain involvement [56,57], both were isolated cases as our patient, and a definite genetic diagnosis was never achieved. 

All proteins participating in the CoQ biosynthesis pathway are nuclear-encoded and are differentially expressed in various tissues and organs, and CoQ content in liver and muscle can fluctuate in response to nutritional or other stressful conditions [58,59]. *ADCK2* was demonstrated here to participate in CoQ biosynthesis and its deficiency in mice worsened physical performance, consistent with muscle dysfunction without behavioral abnormalities [38], resembling the phenotype of the patient. CoQ deficiency is produced as a secondary consequence of defects in components of either oxidative phosphorylation or other mitochondrial pathways [16,17]. There is evidence for tissue-specific expression and function of various members of the *ADCK* family. Mutations in *COQ8A* and *COQ8B* (previously known as *ADCK3* and *ADCK4)* have been shown to induce a decrease of CoQ in the cerebellum and kidney causing cerebellar ataxia and nephrotic syndrome, respectively [15,20,29]. Interestingly, Mfn2 has been demonstrated to contribute to the maintenance of the mitochondrial CoQ pool [13] and regulation of phospholipid transport into mitochondria through mitochondrial-associated membranes [60], in which both Mfn2 and ADCK2 colocalize (Figure S4). Furthermore, yeast strains lacking components of the endoplasmic reticulum–mitochondria encounter structure show destabilization of the CoQ_6_ biosynthesis complex (synthome), leading to impaired synthesis and depletion of mitochondrial CoQ_6_ pool [61]. We found that MEFs depended on Adck2 for the mitochondrial availability of mevalonate-derived isoprenoid side chain of CoQ and cholesterol, which would indicate its role in lipid transport into mitochondria.

Impairment in lipid metabolism associated with defects in both hepatic and skeletal muscle functions were observed in the patient and in *Adck2* +/− mice. Defects in mitochondrial FA oxidation impair physical performance, causing exercise intolerance and reduced strength [62,63]. The mechanism by which *ADCK2* haploinsufficiency alters FA catabolism implicates the accumulation of long- and short-chain fatty acids with lower free carnitine levels as noted in the plasma of the study participant and *Adck2*+/− mice. Substantial alterations in the urinary excretion of metabolites linked to the Krebs cycle and FA β-oxidation were found in mutant mice along with significant reduction in circulating β-hydroxybutyrate, which is derived from β–oxidation. Mice with muscle-specific deletion of acyl-CoA synthetase isoform-1 (*Acsl1M−/−*) or carnitine palmitoyltransferase-1 (*Cpt1bM−/−*) are less physically active and have higher circulating non-esterified FA and mitochondrial dysfunction [64,65]. *Cpt1bM−/−* mice also show poor metabolic adaptation and remodeling of glucose metabolism than when amino acids are used as fuel [64,66]. Similarly, *Acsl1M−/−* mice are severely hypoglycemic and have poor physical endurance combined with an increase in protein catabolism [65,67]. Here, we found that Adck2 deficiency caused defects in pathways of glucose metabolism in the liver and muscle and in MEFs, as shown by plasma lactate accumulation, decrease in respiration, and altered metabolomics profiles. The notion that Adck2 participates in skeletal muscle energy homeostasis was supported by comparative transcriptome and metabolome analyses together with measurement of mitochondrial activities in muscle fibers of WT and *Adck2*+/− mice.

Moreover, we carried out microarray analysis and metabolomics profile in liver to identify genes and metabolites impacted by *Adck2* insufficiency, ultimately leading to hepatic mitochondrial dysfunction. These genes encoded enzymes involved in FA metabolism and Krebs cycle, detoxification pathways, mainly P450 genes, and inflammatory response via interleukin production and JNK pathway activation [68,69]. 

The phenotypes observed in response to *ADCK2* depletion indicated the importance of this gene in pathways involved in mitochondrial FA oxidation and skeletal muscle metabolic regulation. The partial recovery in physical performance combined with the lowering in plasma lactate levels in the II-3 patient and *Adck2*+/− mice upon CoQ supplementation indicated the possibility of improving some of the defects stemming from CoQ deficiency. 

In conclusion, *ADCK2* haploinsufficiency caused an adult onset myopathy with CoQ deficiency, and an overall defect in mitochondrial lipid metabolism, with incomplete penetrance. The heterozygous inactivation of *Adck2* in mouse model recapitulated the human disease. We surmise that ADCK2 participates in the control of mitochondrial FA β-oxidation and CoQ levels in skeletal muscle by affecting the transport of lipids into mitochondria, which is required for both the formation of CoQ isoprenoids side chain and FA oxidation. From a clinical point of view, isolated myopathies and myopathies with lipid accumulation should be tested for this *ADCK2* gene defect because they are likely to be responsive to CoQ supplementation. 

## Supplementary Materials

The following are available online at https://www.mdpi.com/2077-0383/8/9/1374/s1, Figure S1: Laboratory findings in the index patient, Figure S2: Adck2+/− mouse phenotype, Figure S3: Behavioral properties of Adck2+/− mice*,* Figure S4: DCK2 protein is located inside mitochondria*,* Figure S5: Mitochondria properties of mouse tissues, Figure S6: Microarray analysis in mouse skeletal muscle and liver*,* Table S1: Acyl-carnitine analysis in plasma from the index patient II-3, Table S2: Metabolites (mmol/mol creatinine) in urine of Adck2+/− and WT mice. (Reference internal standard ion: undecanodioic, 345), Table S3: Regulated genes in Adck2+/− mouse skeletal muscle, Table S4: Gene ontologies affected by Adck2 haploinsufficiency in mouse muscle, Table S5: Regulated genes in Adck2+/− mouse liver, Table S6: Gene ontologies affected by *Adck2* haploinsufficiency in mouse liver, Table S7: Validation of gene expression analysis by quantitative real-time PCR.
