# Supplementary material for: ADCK2 Haploinsufficiency Reduces Mitochondrial Lipid Oxidation and Causes Myopathy Associated with CoQ Deficiency
Source: J Clin Med. 2019 Sep 2;8(9):1374. doi: 10.3390/jcm8091374 (PMC6780728; doi:10.3390/jcm8091374)
Supplement: Supplementary file 1 [file jcm-08-01374-s001.pdf]

Supplementary information

ADCK2 **haploinsufficiency reduces mitochondrial lipid oxidation and causes myopathy associated with CoQ deficiency..**

Luis Vázquez-Fonseca[1,8,§], Jochen Schäfer[2,§], Ignacio Navas-Enamorado[1,7], Carlos Santos-Ocaña[1,10], Juan D. Hernández-Camacho[1,10], Ignacio Guerra[1], María V. Cascajo[1,10], Ana Sánchez-Cuesta[1,10], Zoltan Horvath[2#], Emilio Siendones[1], Cristina Jou[3,10], Mercedes Casado[3,10], Purificación Gutiérrez[1], Gloria Brea-Calvo[1,10], Guillermo López-Lluch[1,10], Daniel M. Fernández-Ayala[1,10], Ana B. Cortés-Rodríguez[1,10], Juan C. Rodríguez-Aguilera[1,10], Cristiane Matté[4], Antonia Ribes[5,10], Sandra Y. Prieto-Soler[6], Eduardo Dominguez-del-Toro[6], Andrea di Francesco[8], Miguel A. Aon[8], Michel Bernier[8], Leonardo Salviati[9], Rafael Artuch[3,10], Rafael de Cabo[8], Sandra Jackson[2] and Plácido Navas[1,10]

Supplementary Results

Case report

The male index patient (subject II-3, Fig. S1A) presented to our clinic at 45 years of age with a 15-year history of slowly progressive muscle weakness and myalgia, which occurred at rest but worsened with exercise. Past medical history was unremarkable except for renal disease of unknown cause in childhood, which spontaneously improved. Family history was negative for neurological disease. On examination, moderate proximal symmetrical myopathy, more pronounced in the arms, was noted and the patient was unable to lift his arms above the horizontal position. The patient had a hyperlordotic, waddling gait and was only able to walk 100 meters without the aid of crutches. Bilateral scapular winging was present, and bilateral atrophy of the biceps, triceps, and quadriceps was noted, whilst the deltoid muscles were well preserved. Calf hypertrophy was present. The Trendelenburg sign was positive, and the patient was unable to rise from squatting. Pulmonary function was mildly impaired (vital capacity was 85 % of normal). Nerve conduction studies did not reveal a significant polyneuropathy. There were no central neurological deficits, bulbar or ocular problems and no ataxia; cognitive function was normal. On EMG examination, a myopathic pattern was found, with copious spontaneous activity (fibrillation, myotonic discharges and complex-repetitive discharges). Echocardiogram was normal. Abdominal ultrasound examination revealed liver steatosis. MR spectroscopic imaging of the brain showed multifocal elevation of lactate, alanine, lipids and free macromolecules. A muscle biopsy performed 5 years earlier showed moderate myopathic changes with marked lipid accumulation, consistent with a lipid storage myopathy. No ragged red fibers or inflammatory changes were identified. Total carnitine was mildly reduced in muscle and a diagnosis of carnitine deficiency was made. The patient had received carnitine replacement therapy (4 g of carnitine per day) since this time without any improvement in his condition, which had continued to worsen over this time at increased speed.

Pathological laboratory findings at presentation included creatine kinase (CK) activity of 13.8 µKat/s (normal <3.2), urea 1.7 mM (normal 3.2-7.3), lactate 6.5 mM (normal range 0.5-2.2), LDH 10.3 µmol/s (<3.8), ALAT 0.96 µmol/s (<0.8), ASAT 1.5 µmol/s (<0.8), γ-GT 1.4 µmol/s (<0.8), GLDH 0.28 µmol/s (<0.12), and myoglobin 238 µg/L (< 7.2). Plasma acyl-carnitine analysis by tandem mass spectroscopy identified moderately elevated levels of saturated short- and medium-chain length acyl-carnitine species (Table S1, column 1), compatible with multiple acyl-CoA dehydrogenase deficiency (MADD). Plasma free carnitine (on carnitine supplementation) was within the normal range (57.8 µmol/L; reference range 20-70 µmol/L). Urinary organic acid analysis revealed a 3-fold elevation of ethylmalonic acid with normal amounts of lactate and pyruvate. The plasma acyl-carnitine and lactate levels were normal in subjects I-1 and II-2.

Riboflavin therapy (200 mg per day) was initiated immediately based upon these findings, and two months later a further muscle biopsy was performed. Histological examination revealed some fiber atrophy and small vacuoles in many fibers (Fig. 1A), with lipid droplets apparent in some fibres with the oil-red-O stain (Fig. 1A). Only occasional ragged-red fibres were noted with the Gomori trichrome stain (not shown).

On riboflavin therapy, the plasma acyl-carnitine profile began to normalize. After 2 months, the levels of hexanoylcarnitine were within the 99 percentile limit of the normal range, while the C4, C8 and C10 species where still mildly elevated (Table S1). The plasma acyl-carnitine profile was further improved after 6 months (timepoint 4, Table S1), and this was accompanied by a moderate reduction in the levels of plasma lactate, CK, myoglobin, and total LDH (Fig. S1E). After 6 months of combined carnitine/riboflavin therapy, there was no improvement in the patient's symptoms, and he now required opiates for severe myalgia, which had worsened during this time. There was no change in the EMG, but MRS showed an improvement in cellular lactate and lipid levels in the brain, indicating a decrease in membrane destruction. As riboflavin therapy did not appear to be having an effect on disease progression, supplementation with CoQ_{10} in the form of nanoparticles in liquid suspension was

recommended (initial dose, 75 mg CoQ per day; Fig. S1E). His clinical condition was unchanged after 4 months of combined carnitine/riboflavin/CoQ therapy, but there was a slight improvement in the plasma lactate and myoglobin levels (Fig. S1E). One month later, the patient stopped taking $CoQ_{10,}$ but after 3 weeks, his pain worsened, his muscle strength decreased, and he was unable to lift his arms, indicating that the symptoms were partially dependent on $CoQ_{10.}$ After 4 months, the patient resumed CoQ_{10} at a reduced dose (35 mg per day), but his muscle strength continued to diminish and the EMG results were unchanged, so the dose of CoQ_{10} was increased (Fig. S1E). As the C4, C6, C8 and C10 acyl-carnitines were still elevated (Table S1, time point 5), and riboflavin and carnitine supplementation were without effect on the clinical course, both riboflavin and carnitine were stopped after ~18 months.

The patient's clinical symptoms progressively worsened, so that 3 years after he first presented to us he could only walk 50 m with the aid of crutches or a walker and within 1 year this was reduced to 20 m and he could no longer rise from the sitting position. Nerve conduction studies performed at this time revealed a mild axonal polyneuropathy. One year later, walking distance was reduced to 10 m and non-invasive ventilation was initiated because of global respiratory failure. Measurement of testosterone in plasma at this time revealed low levels of both total (patient 5.44; range 9.08-55.23 nmol/L) and free (18.1; range 25.0-80.0 nmol/L) testosterone. The levels of LSH (<0.5; range 2.0-12.0 units/L), FSH (<0.4; range 1.0-8.0 units/L), and cortisol (91; normal range 125-667nmol/L) were also reduced whilst that of DHEAS was increased (11.9; range 1.9-8.41 units/L). A diagnosis of primary partial pituitary failure, involving the gonadotropic, corticotropic, and somatotropic axes was made. Vitamin D deficiency was also diagnosed, with reduced levels of 25-OH-Vitamin D (<4, deficiency classed as less than 10 ng/mL) and 1,25-OH-vitamin D (13.6, normal 19.2-60 pg/mL). The patient was prescribed testosterone (50 mg/day patch), hydrocortisone (10 mg/day), and vitamin D (Fig. S1E). The patient reported an improvement in his feeling of well-being. Despite these interventions, eight years after presentation, the patient could not stand freely, required a wheel chair~~,~~ and help with all

activities of daily living. A muscle MRI performed 2 years later revealed severe fatty degeneration of the shoulder girdle, deltoid, biceps, hamstring and calf muscles, whilst the triceps were well preserved (Fig. S1B). A biopsy of the triceps revealed some cox-negative and ragged red-fibres, but no lipid storage. Paracrystalline inclusions were observed in approximately one quarter of the mitochondria (not shown). Treatment with ubiquinol, 150 mg/per day (GeriMed, GmBH), was initiated 10 years after presentation, and was associated with stabilization of the plasma levels of lactate, CK, myoglobin, and LDH, which returned to normal or near normal values. The levels of C8 and C10 acyl-carnitines in the plasma remained moderately elevated. Twelve years after he presented, the patient died of bolus aspiration at the age of 57 due to newly developed dysphagia.

As the muscle biopsy findings were compatible with a mitochondrial disorder, we measured the activity of the mitochondrial respiratory chain complexes in muscle homogenate from the patient. The activities of complexes I, III, and IV compared to the activity of citrate synthase, a mitochondrial marker, were within the control range, whilst the combined activities of complexes I and III, which requires the participation of endogenous CoQ, was reduced (Fig. 1H), indicative of a CoQ deficiency. The combined activities of complex II+III were also reduced in cultured fibroblasts from both the index patient (ADCK2-II-3 in Fig. 1I) and his asymptomatic sister, II-2 (Fig. 1I). Accordingly, CoQ levels were significantly reduced in cultured fibroblasts from the index patient and subject II-2 compared to control fibroblast cultures (Fig 1E). Sequencing of the entire mitochondrial genome in the patient did not reveal any pathogenic mutations (not shown).

Plasma lactate levels were elevated in the index patient, but normal in subject II-2. We next investigated lactate production in cultured fibroblasts from both individuals. Fibroblasts (25 x 10^3 cells/mL) from controls and subjects II-3 and II-2 were seeded and grown for 6 days in glucose-rich medium, and then the lactate concentration was measured in the culture medium. Lactate production was elevated in cultured fibroblast from both the index patient and his sister compared with that in control fibroblasts (control: 0.050±0.003 nmol/cell; II-2: 0.350±0.005 nmol/cell; II-3: 0.290±0.009

nmol/cell; $p<0.002$; n=4).

A truncating mutation in ADCK2 identified in the patient and subject II-2.

As the clinical findings were strongly indicative of MADD, we sequenced *ETFDH*, *ETFA*, and *EDFB* in DNA from the index patient, II-3. No mutations were identified in any of these genes. Next, targeted sequencing of the genes known or predicted to be involved in CoQ biosynthesis was performed in DNA from the patient. No mutations were detected in the genes *ADCK1*, *ADCK3, ADCK4*, *ADCK5*, *PDSS1*, *PDSS2*, *COQ2*, *COQ3*, *COQ4*, *COQ5*, *COQ6*, *COQ7*, *COQ8A*, *COQ8B, COQ9*, *COQ10A*, *COQ10B* or *PPTC7*. A single, heterozygous c.997C>T p.Arg333* sequence change was identified in exon 2 of the gene *ADCK2* (NM_052853.3) (Fig. S1C and S1D). This sequence change is predicted to result in the replacement of arginine with a premature termination codon at amino acid position 333 in the encoded protein. This sequence change is absent among the ~ 13005 alleles listed on the NHLBI exome sequencing project exome variant server (http://evs.gs.washington.edu/EVS/) and is not listed on the 1000 genomes project database, nor in the ExAC database (http://exac.broadinstitute.org). Screening for the c.997C>T sequence change in other family members revealed that the patient's clinically unaffected mother, subject I-1, and sister, subject II-2, were heterozygous for this sequence change, whilst the other sibling, subject II-1, does not harbor this change (Figs. S1C and S1D). These results were confirmed by mitochondrial panel sequencing and no other sequence changes were detected upon sequencing of the cDNA obtained from the index patient.

The c.997C>T mutation resides 83 nucleotides upstream of an exon junction in exon 2 of the 8 exon gene, and thus would be predicted to lead to the rapid decay of the affected mRNA. Extraction of RNA from blood and cultured fibroblasts from the index patient and individual II-2, followed by reverse transcription and subsequent sequencing of the cDNA, revealed decreased abundance of mRNA harboring the premature translation-termination codon arising from the c.997C>T mutation, confirming that this mRNA species undergoes non-sense mediated decay in these tissues (Fig. S1C).

Further evidence for this is provided by the finding that the amount of *ADCK2* mRNA (Fig. 1B) and protein (Figs. 1C and 1D) is significantly decreased in cultured fibroblasts from the index patient and individual II-2. To understand the role of this mutation in the level of CoQ in patient' fibroblasts, we transformed patient's cells with the plasmid pRRL harboring the WT allele of *ADCK2*. Figures 1F and 1G shows that these cells increased both mRNA and CoQ content.

Supplementary Table 1. Acyl-carnitine analysis in plasma from the index patient II-3.

**Acyl-carnitine species**	2004 + carnitine	2004 carnitine + riboflavin 1 month	2004 carnitine + riboflavin 2 months	**4**	**5**	**6**	**7**	**8**	**9**	**10**	**11**	**12**	**13**	**14**	**15**	**16**	**17**
**C4**	**2.7**	**2.2**	1.3	1.0	**2.4**	**1.7**	**2.3**	0.7	1.0	1.6	**2.2**	**1.9**	1.1	1.2	1.1	1.0	0.9
**C5**	**1.9**	**2.3**	1.4	1.3	**1.9**	**2.0**	**1.8**	0.9	0.9	1.6	1.4	0.8	1.1	1.8	2.3	2.1	2.9
**C5:1**	0.2	0.2	0.2	0.2	0.2	0.2	0.1	0.1	0.1	0.1			0.2	0.1	0.2	0.1	0.1
**C6**	**2.1**	1.2	0.8	0.9	1.1	**1.5**	**1.8**	0.6	1.0	0.8	**1.7**	1.3	0.8	1.3	1.1	1.3	1.1
**C8**	**3.4**	**1.9**	**1.5**	1.3	**2.1**	**2.2**	**2.7**	**1.7**	1.2	1.1	**4.0**	**1.8**	**1.7**	**2.5**	**2.1**	**2.3**	**1.5**
**C10**	**2.8**	**1.8**	**1.7**	1.3	**2.1**	**2.3**	**2.3**	**1.4**	1.2	1.0	**3.9**	**2.0**	**1.6**	**3.2**	**1.7**	**1.7**	1.3
**C10:1**	1.1	0.9	0.7	0.4	0.9	1.3	1.3	0.7	0.7	0.7	**1.7**	0.7	0.7	1.1	0.8	0.7	0.7
**C12**	0.5	0.6	0.4	0.4	0.5	0.4	0.7	0.3	0.4	0.4	0.6	0.6	0.4	0.4	0.4	0.4	0.4
**C12:1**	**1.6**	1.0	0.9	0.8	0.9	0.9	**1.7**	0.7	0.4	0.4	**2.2**	0.7	1.2	1.5	1.3	0.9	0.6
**C14**	0.5	0.6	0.9	0.4	0.6	0.3	-	0.5	0.3	0.4	0.4	0.5	0.4	0.3	0.3	0.3	0.3
**C14:1**	1.0	0.8	0.9	0.7	0.4	0.5	1.0	0.4	0.3	0.4	0.9	0.4	0.6	0.6	0.6	0.3	0.3
**C16**	0.6	0.6	0.5	0.6	0.8	0.5	0.9	0.6	0.5	0.5	0.6	0.5	0.7	0.5	0.4	0.5	0.4
**C18**	0.2	0.3	0.2	0.4	0.4	0.3	0.3	0.3	0.2	0.3	0.3	0.3	0.3	0.3	0.2	0.2	0.2

The values shown are the fold increase of the measured value over the 99-percentile value for adults. The numbers shown in the upper column (4-17) refer to the time points indicated in panel C of Figure S1E. Bold type represents significant differences.

Supplementary Table 2. Metabolites (mmol/mol creatinine) in urine of *Adck2*+/- and WT mice. (Reference internal standard ion: undecanodioic, 345).

Data were analyzed via Student's t test. Data represent SD *P < 0.01, **P < 0.05 vs. mutant (n=5)

**Metabolite (Ion)**	***Adck2+/-***	**WT**
Lactate (219)	7.7±0.2	2.3±0.03*
Malate (233)	24.8±3	13.7±0.5**
Fumarate (245)	341.0±25	182.8±18**
α-Ketoglutarate (347)	542.0±33	315.8±21*
Succinate (247)	236.8±26	148.4±22*
Glutarate (261)	78.1±20	54.6±16
Suberic acid (303)	20.4±5	13.3±3
Adipic acid (275)	31.6±6	15.0±2*
Ethylmalonic acid (217)	12.5±1	7.5±0.7**
Hexanoylglycine (230)	29.9±9	18.3±4**

Supplementary Table 3. Regulated genes in *Adck2*+/- mouse skeletal muscle.

Symbol	Z ratio	Fold Change	Gene description and function	Disease-related information
Xlr4a	-8.6	-2.6	Synaptonemal Complex Protein 3 (Spermatogenesis)	
Rps26	-6.1	-2.0	Ribosomal Protein S26	
Anapc13	-6.0	-1.9	Cyclosome (APC/C), subunit 13	
Mrpl52	-5.9	-1.9	Mitochondrial Ribosomal Protein L52	
Grcc10	-5.5	-1.8	Unknown protein-coding gene	
Fxyd1	-5.5	-1.8	FXYD Domain Containing Ion Transport Regulator 1 (Muscle contraction)	
Polr2i	-5.5	-1.8	Polymerase (RNA) II	
Lgals1	-5.5	-1.8	Lectin, Galactoside-Binding, Soluble, 1 (Galectin 1)	Autocrine negative growth factor that regulates cell proliferation and induces apoptosis
ROMO1	-5.4	-1.8	Reactive Oxygen Species Modulator 1	Induces ROS production necessary for cell proliferation and coordination of mitochondrial morphology and cell proliferation
Rpl28	-5.4	-1.8	Ribosomal Protein S28	
Phpt1	-5.4	-1.8	Phosphohistidine Phosphatase 1 (KCa3.1 channel inhibitor)	
Pdlim5	6.2	2.0	PDZ and LIM Domain 5 (scaffold protein that tethers protein kinases to the Z-disk in striated muscles)	Overexpression promotes the development of heart hypertrophy
Ttid	6.3	1.9	Myotilin (Myofibril assembly and stability at the Z lines in muscle cells)	Overexpressed in myofibrillar myopathies and in response to muscular dystrophy
Cbfb	6.3	2.1	Core-Binding Factor beta Subunit (Hematopoiesis- and osteogenesis-specific transcription factor)	
Fbxo32	6.5	2.2	F-Box Protein 32 (Component of SCF ubiquitin-protein ligase complex)	Binds to phosphorylated target proteins during skeletal muscle atrophy (highly expressed during muscle atrophy, whereas mice deficient in this gene were found to be resistant to atrophy)
Ppp1cb	6.6	2.0	Protein Phosphatase 1 (Cell division, glycogen metabolism, muscle contractility and protein synthesis)	
Rbbp7	6.6	2.1	Retinoblastoma Binding Protein 7 (Histone deacetylase complexes for gene repression by epigenetic modification)	
Lmcd1	6.7	1.9	LIM and Cysteine Rich Domains 1 (Transcriptional repressor of GATA6-mediated expression, muscle-specific)	Involved in activation of mitophagy in response to mitochondrial depolarization
Srp54	6.7	2.2	Signal Recognition Particle 54kDa	

			(Binds to pre-secretory proteins)	
Car3	7.5	2.2	Carbonic Anhydrase 3 (Skeletal muscle specific)	Duchenne muscle dystrophy have a higher CA3 level
2310032D16Rik	8.6	2.7	Glycerophosphocholine Phosphodiesterase GDE1<br>(Skeletal muscle development)	

The functional description of each gene was updated from the GeneCard of The Human Gene Compendium (Weizmann Institute of Science), http://www.genecards.org/, and from the Mouse Genome Database (MGD), Mouse Genome Informatics, The Jackson Laboratory, Bar Harbor, Maine, http://www.informatics.jax.org/.

Supplementary Table 4. Gene Ontologies affected by *Adck2* haploinsufficiency in mouse muscle.

Gene Ontology Term	Zscore
GO0006099 TRICARBOXYLIC ACID CYCLE	5.8
GO0016627 CYTOSOLIC OXIDOREDUCTASE	5.5
GO0006635 FATTY ACID BETA OXIDATION	5.4
GO0003995 ACYL COA DEHYDROGENASE ACTIVITY	5.3
GO0000163 PROTEIN PHOSPHATASE TYPE 1 ACTIVITY	5.3
GO0005759 MITOCHONDRIAL MATRIX	5.2
GO0046872 METAL ION BINDING	4.6
GO0016874 LIGASE ACTIVITY	4.6
GO0050660 FAD BINDING	4.5
GO0004842 UBIQUITIN PROTEIN LIGASE ACTIVITY	4.4
GO0030145 MANGANESE ION BINDING	4.3
GO0017111 NUCLEOSIDE TRIPHOSPHATASE ACTIVITY	4.3
GO0000287 MAGNESIUM ION BINDING	4.1
GO0015071 PROTEIN PHOSPHATASE TYPE 2C ACTIVITY	4.1
GO0004872 RECEPTOR ACTIVITY	4.0
GO0004722 PROTEIN SERINE OR THREONINE PHOSPHATASE	3.8
GO0005604 BASEMENT MEMBRANE	-3.9
GO0007001 CHROMOSOME ORGANIZATION AND BIOGENESIS	-4.2
GO0005581 COLLAGEN	-4.8
GO0030020 EXTRACELLULAR MATRIX (STRUCTURAL)	-4.9
GO0005762 MITOCHONDRIAL LARGE RIBOSOMAL SUBUNIT	-4.9
GO0003899 DNA DIRECTED RNA POLYMERASE ACTIVITY	-5.0
GO0005843 CYTOSOLIC SMALL RIBOSOMAL SUBUNIT	-6.3
GO0016071 MRNA METABOLIC PROCESS	-7.0
GO0003954 NADH DEHYDROGENASE ACTIVITY	-7.2
GO0004129 CYTOCHROME C OXIDASE ACTIVITY	-7.7
GO0042254 RIBOSOME BIOGENESIS AND ASSEMBLY	-8.0
GO0005830 CYTOSOLIC RIBOSOME	-10.5
GO0008137 NADH DEHYDROGENASE (UBIQUINONE) ACTIVITY	-11.0
GO0006412 TRANSLATION	-11.9
GO0003735 STRUCTURAL CONSTITUENT OF RIBOSOME	-18.7

Supplementary Table 5. Regulated genes in *Adck2*+/- mouse liver.

Symbol	Zratio	Fold Change	Gene description and function	Disease-related information
Cyp4a14	4.5	2.3	Cytochrome P450	
Cyp2b9	3.9	1.8	Cytochrome P450	
Cyp2b20	3.8	1.9	Cytochrome P450	
Mvd	3.4	2.2	Mevalonate Pyrophosphate Decarboxylase (cholesterol metabolism)	
Tmie	3.2	1.6	Transmembrane inner ear protein	
Psen2	3.1	1.5	Presenilin 2 (Notch receptor, PI-3K cascade and signaling by FGFR)	• Alzheimer<br>• Mental retardation<br>• Nervous system development
Gstt3	2.9	1.6	Glutathione S-transferase theta 3	
Vldlr	2.9	1.7	Very Low Density Lipoprotein Receptor	
Pte2a (Acot2)	2.8	2.3	Mitochondrial Acyl-CoA Thioesterase 1	
Pgpep1	2.8	1.5	Pyroglutamyl-Peptidase (Glyoxylate and dicarboxylate metabolism)	
Rasl11b	2.8	1.7	Small GTPase RAS-L11B	
EG624219	2.7	2.2	Fat Cell Differentiation Protein (triglyceride homeostasis)	
Sbk	2.6	1.8	Serine/Threonine-Protein Kinase	
BC013476	2.5	2.2	Cytochrome P450	
Olig1	2.5	1.5	Oligodendrocyte Transcription Factor 1	
Dnmt3b	2.5	1.7	DNA Methyltransferase HsaIIIB	
Mmp14	2.5	1.6	Matrix Metallopeptidase 14	
Cldn5	2.5	1.5	Claudin 5 (tight junction)	
Gstt2	2.4	1.5	Glutathione *S*-Transferase Theta 2	
Elovl1	2.3	1.5	Fatty Acid Elongase 1	
Dusp6	2.3	1.5	Mitogen-Activated Protein Kinase Phosphatase	
Adamts7	2.3	1.5	Disintegrin metallopeptidase	Activated in mitophagy in response to mitochondrial depolarization
Ly6a	2.3	1.5	Lymphocyte Antigen 6 Complex	
Mgst3	2.3	1.6	Microsomal Glutathione *S*-Transferase	
Srebp2	2.2	1.5	Sterol Regulatory Element Binding Transcription factor 2	Activates transcription of LDL receptor, cholesterol and fatty acid
Fasn	2.1	1.7	Fatty Acid Synthase (short-chain)	Obesity and trophoblastic neoplasm
Aacs	2.1	2.0	Acetoacetyl-CoA Synthetase (Involved in utilizing ketone body for fatty acid synthesis)	
Chrna4	2.1	2.3	Plasma membrane sodium channel	Overexpressed in liver cancer ESC pluripotency maintenance
Pte2b (Acot4)	2.1	1.5	Peroxisomal Acyl-CoA Thioesterase 2B	
Pdk4	2.1	2.1	Pyruvate Dehydrogenase Kinase 4	ESC pluripotency maintenance
Crat	2.1	1.5	Carnitine O-acetyltransferase (short-chain fatty acid specific)	
Acot1	2.1	1.6	Acyl-CoA thioesterase (Fatty Acid Synthase)	
Gstp1	-3.5	-1.7	Glutathione S-Transferase Pi 1	

Supplementary Table 6. Gene Ontologies affected by *Adck2* haploinsufficiency in mouse liver.

Gene Ontology Term		p-value	Enrichment [1]
GO:0004312	Fatty acid synthase activity	1.1E-4	122.56
GO:0016290	Palmitoyl-CoA hydrolase activity	5.24E-6	85.80
GO:0006084	Acetyl-CoA metabolic process	1.3E-5	64.35
GO:0006637	Acyl-CoA metabolic process	4.02E-11	54.60
GO:0004602	Glutathione peroxidase activity	6.23E-4	53.62
GO:0004364	Glutathione transferase activity	2.6E-5	51.48
GO:0016790	Thiolester hydrolase activity	4.24E-8	51.07
GO:0047617	Acyl-CoA hydrolase activity	3.3E-5	47.66
GO:0000038	Very long-chain fatty acid metabolic process	4.11E-5	44.38
GO:0001676	Long-chain fatty acid metabolic process	4.14E-8	30.28
GO:0005777	Peroxisome	7.05E-6	18.49
GO:0006631	Fatty acid metabolic process	2.25E-11	17.16
GO:0008203	Cholesterol metabolic process	9.81E-4	15.32
GO:0006732	Coenzyme metabolic process	3.42E-7	15.09
GO:0032787	Monocarboxylic acid metabolic process	8.05E-11	12.68
GO:0008202	Steroid metabolic process	7.81E-4	9.59
GO:0043436	Oxoacid metabolic process	3.93E-9	7.87
GO:0006082	Organic acid metabolic process	5.14E-9	7.69
GO:0016491	Oxidoreductase activity	7.7E-4	4.52
GO:0032692	Negative regulation of IL-1 production	9.32E-4	-1072.44
GO:0032682	Negative regulation of chemokine production	8.16E-4	-1225.64
GO:0043508	Negative regulation of JUN kinase activity	7.58E-4	-1319.92
GO:0032691	Negative regulation of IL-1 beta production	7.58E-4	-1319.92
GO:0002674	Negative regulation of inflammatory response	6.99E-4	-1429.92
GO:0032930	Positive regulation of superoxide generation	6.99E-4	-1429.92
GO:0008432	JUN kinase binding	6.41E-4	-1559.91
GO:0051771	Negative regulation of nitric oxide synthesis	3.5E-4	-2859.83
GO:0097057	TRAF2-GSTP1 complex	1.75E-4	-5719.67
GO:2000469	Negative regulation of peroxidase activity	1.17E-4	-8579.50

[1] Gene Ontology Enrichment represents the selection of genes associated with a specific GO. It is calculated using GORILLA software (Gene Ontology enrichment analysis and visualization tool at http://cbl-gorilla.cs.technion.ac.il/). Enrichment is calculated as follows: E=(b/n)/(B/N), being "N" the total number of annotated genes in the mouse genome, "B" the total number of genes associated with a particular GO, "n" the number of regulated genes in the *Adck2*+/- mouse and "b" the number of regulated genes in the *Adck2*+/- mouse associated with a particular GO.

Supplementary Table 7. Validation of gene expression analysis by quantitative real-time PCR.

	LIVER		MUSCLE	
	Fold change			
Gene symbol	Array	qPCR	Array	qPCR
Apoa2	1.03	n.d.	1.75	4.35 ± 2.43
Cdx4	1.10	1.20 ± 0.09	1.14	2.30 ± 0.16
Ctsh	1.43	1.15 ± 0.21	1.02	1.92 ± 0.12
Gstp1	-1.60	-1.33 ± 0.06	-1.33	-1.34 ± 0.07
Mia2	1.11	2.37 ± 0.80	1.07	1.79 ± 0.20
Nduf2b	-1.09	-1.39 ± 0.07	-1.79	-1.48 ± 0.07
Phlda2	1.12	2.43 ± 0.80	1.10	1.26 ± 0.22
Slc2a2	1.19	2.27 ± 0.22	1.12	4.69 ± 1.5
Tomm7	1.11	1.58 ± 0.05	-1.76	-1.96 ± 0.11
Trf	1.01	3.86 ± 2.83	1.51	4.19 ± 0.89

Quantitative real-time PCR (qPCR) of selected genes whose expression was affected by the *Adck2* haploinsufficiency. Values represent the fold change in gene expression of both liver and muscle genes in *Adck2+/-* mice relative to wild type animals. Not detected (n.d.)

Supplementary figures

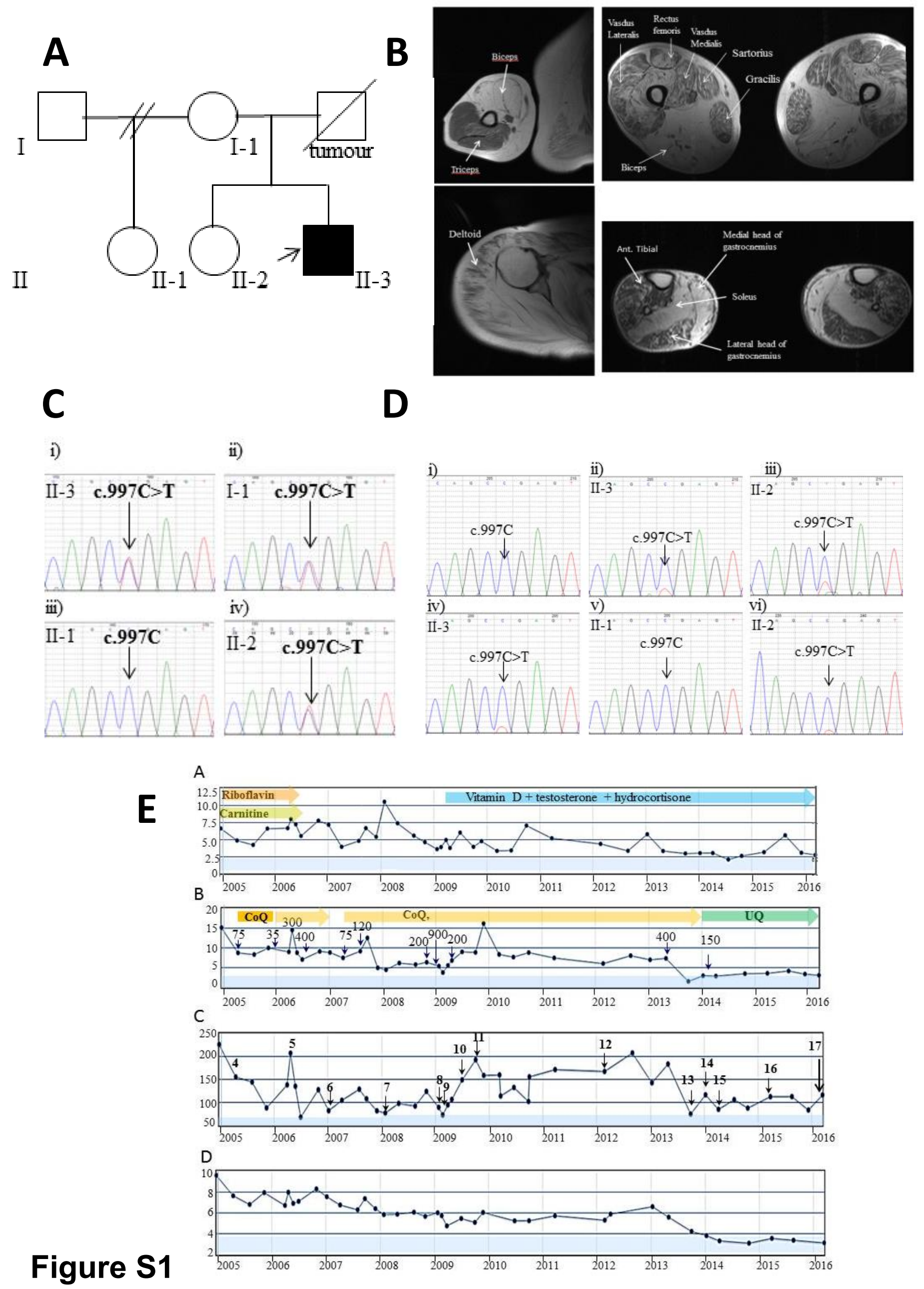

Figure S1

Supplementary Figure 1related to Fig.1. Laboratory findings in the index patient. A. Pedigree of the index patient, subject II-3. B. ADCK2 deficiency is associated with selective muscular fatty degeneration in the index patient. TI-weighted skeletal muscle MRI of the right arm showing pronounced fatty degeneration of the biceps, but not the triceps; the right deltoid showing peripheral sparing and central involvement; the thighs, showing preferential degeneration of the hamstring muscles, the lower legs, showing preferential degeneration of the soleus and the medial head of the gastrocnemius. C. Sequencing chromatograms showing the heterozygous c.997C>T sequence change in i) blood from the index patient II-3, ii) his mother I-1, iv) his sister, II-2, and iii) wild type sequence in blood from his sister II-1. D. Sequence chromatograms obtained following reverse transcription of RNA isolated from blood (i-iii) and fibroblasts (iv-vi) of family members and subsequent sequencing of the cDNA. Note that only a small peak of c.997C>T is apparent in mRNA isolated from blood and fibroblasts of II-3 (ii and iv) and II-2 (iii and vi) indicating that the allele harboring this mutation has undergone nonsense-mediated decay. Only wild type sequence was

visible in cDNA from control blood (i) and fibroblasts of II-1 (v). E. Findings in plasma of patient II-3 and schematic of the treatment regime. A: Plasma lactate levels (reference values 0.5-2.2 mmol/L). The time frame of riboflavin, carnitine, vitamin D, testosterone and hydrocortisone treatment is indicated. B: Creatine kinase activity values (normal values (<3.2 µmol/s). The time frame of CoQ or ubiquinone treatment is shown. The amount of CoQ or ubiquinone administered per day (mg) is indicated. C: Myoglobin (normal values <58 µg/L). The time points at which plasma acyl-carnitine analysis was performed is indicated in this panel (4-17), and corresponds to the column headings in Table S7. D: Total LDH activity (reference values 2.25-3.75 µmol/s). The normal values are indicated by blue shading in each panel.

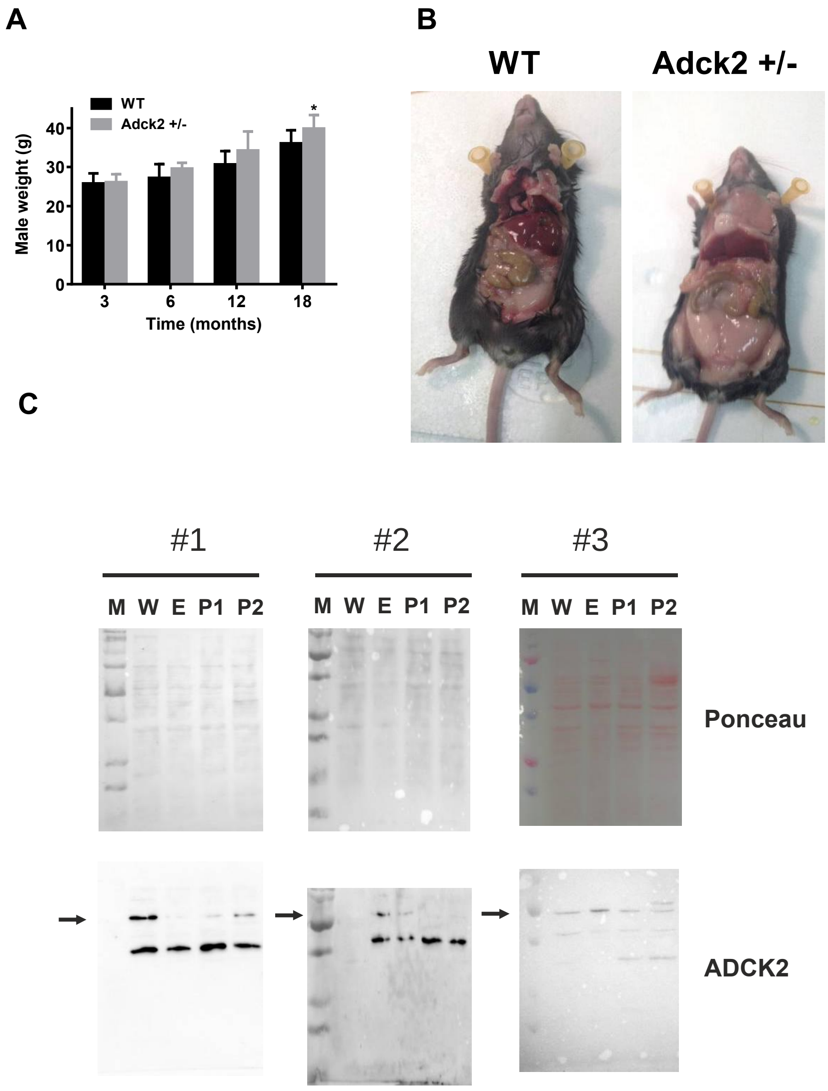

Figure S2

Supplementary Figure 2 related to Figs. 1 and 2. *Adck2+/- mouse phenotype.* (A) Weight trajectories of WT and *Adck2*+/- male mice on standard diet. Bars represent mean + SD. Data were analyzed by Student's t test. * p≤0.05 vs. 18-month-old WT mice; n=15. (B) Representative images of WT and *Adck2*+/- mice at 18 months of age. (C) Ponceau staining of nitrocellulose membranes corresponding to the western blot of Figure 1C.

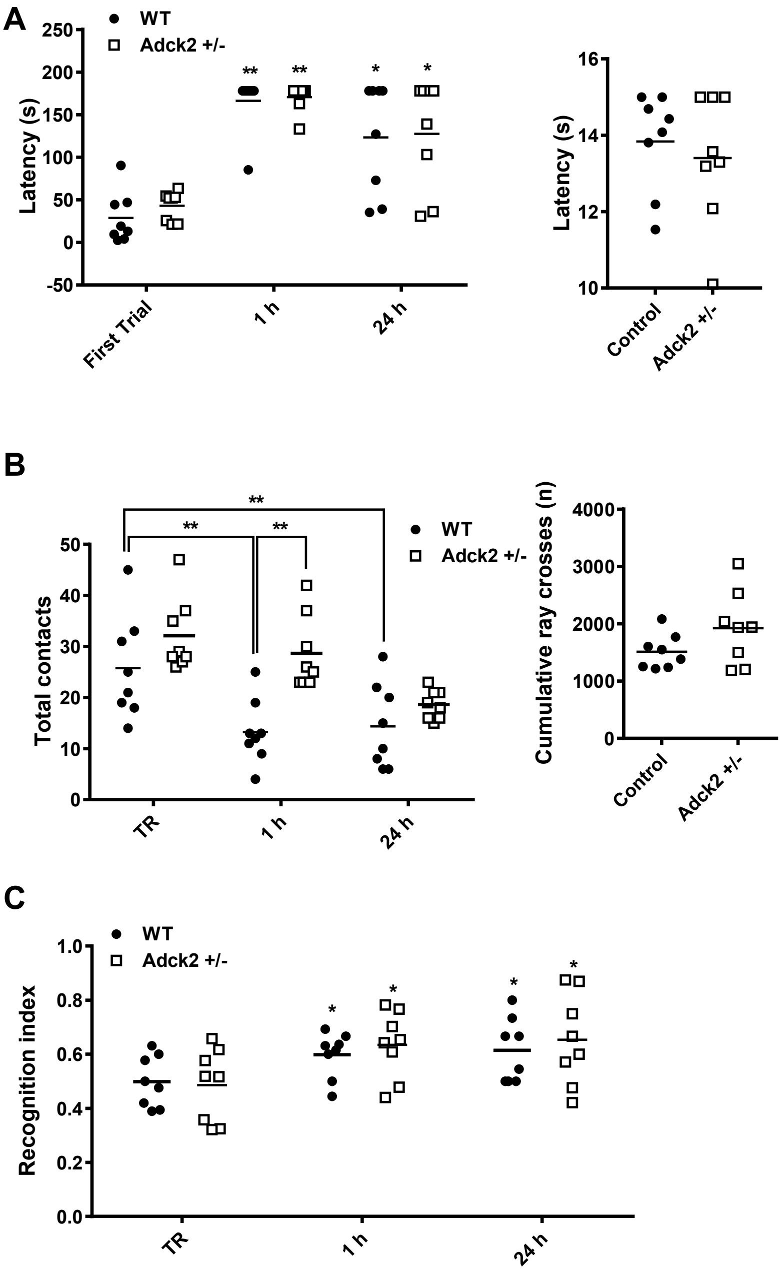

Figure S3

Supplementary Figure 3 related to Fig.2. *Behavioral properties of Adck2+/- mice.* (A) Emotional memory test of WT and *Adck2*+/- mice during the first trial and after 1 and 24 hours post-trial (left panel). Pain sensitivity was determined with the hot-plate test (right panel). (B) Exploratory activity was determined during Novel Object Recognition (NOR) task by measuring the total number of object contacts during training (TR) and after 1 and 24 hours post-TR (left panel). General exploratory activity in the open field apparatus was also monitored (right panel). C. Behavioral properties were analyzed by the novel object recognition index. *$p \leq 0.05$ vs. first trial; **$p \leq 0.01$ vs. WT (n=16). Data were analyzed by 1-way ANOVA (left panels) or Student's t test (right panels).

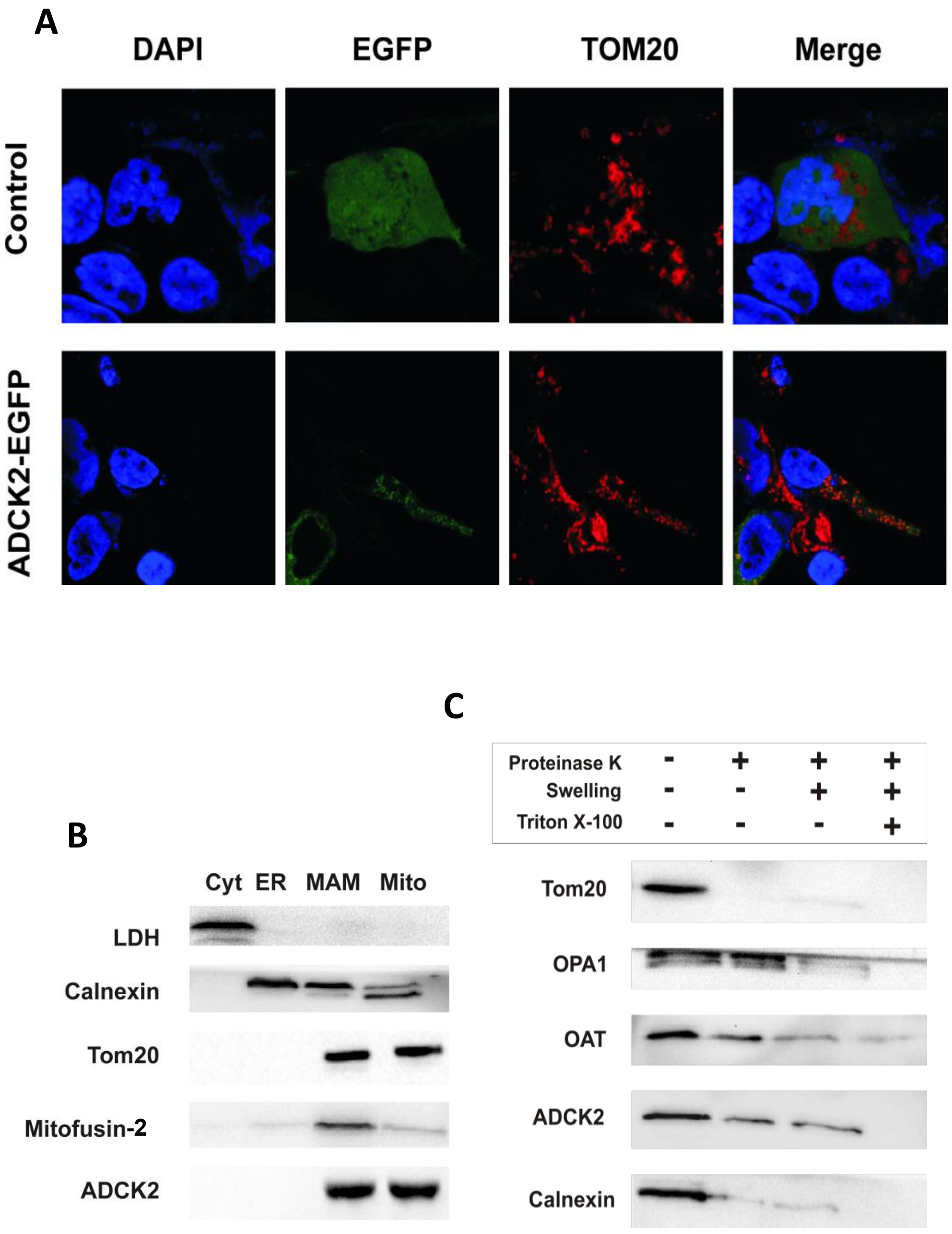

Figure S4

Supplementary Figure 4 related to Fig. 3. ADCK2 *protein is located inside mitochondria*. A. Co-immunolocalization of cytochrome c and endogenous ADCK2 protein in HEK293 cells by confocal microscopy. B. Subcellular fractions from HEK293 cells were immunoblotted for ADCK2, lactate dehydrogenase (LDH), calnexin, TOM20, and mitofusin-2. Cyt, cytosol; ER, endoplasmic reticulum; MAM, mitochondria-associated membranes; Mito, mitochondria. Note the enrichment of ADCK2 in MAM and mitochondrial fractions. C. Mitochondrial-enriched fractions from HEK293 cells were treated with proteinase K combined with swelling and Triton X-100. Immunoblotting was then performed with the indicated primary antibodies. TOM20, outer membrane marker; calnexin and OPA1, intermembrane space markers; ornithine aminotransferase (OAT), matrix marker.

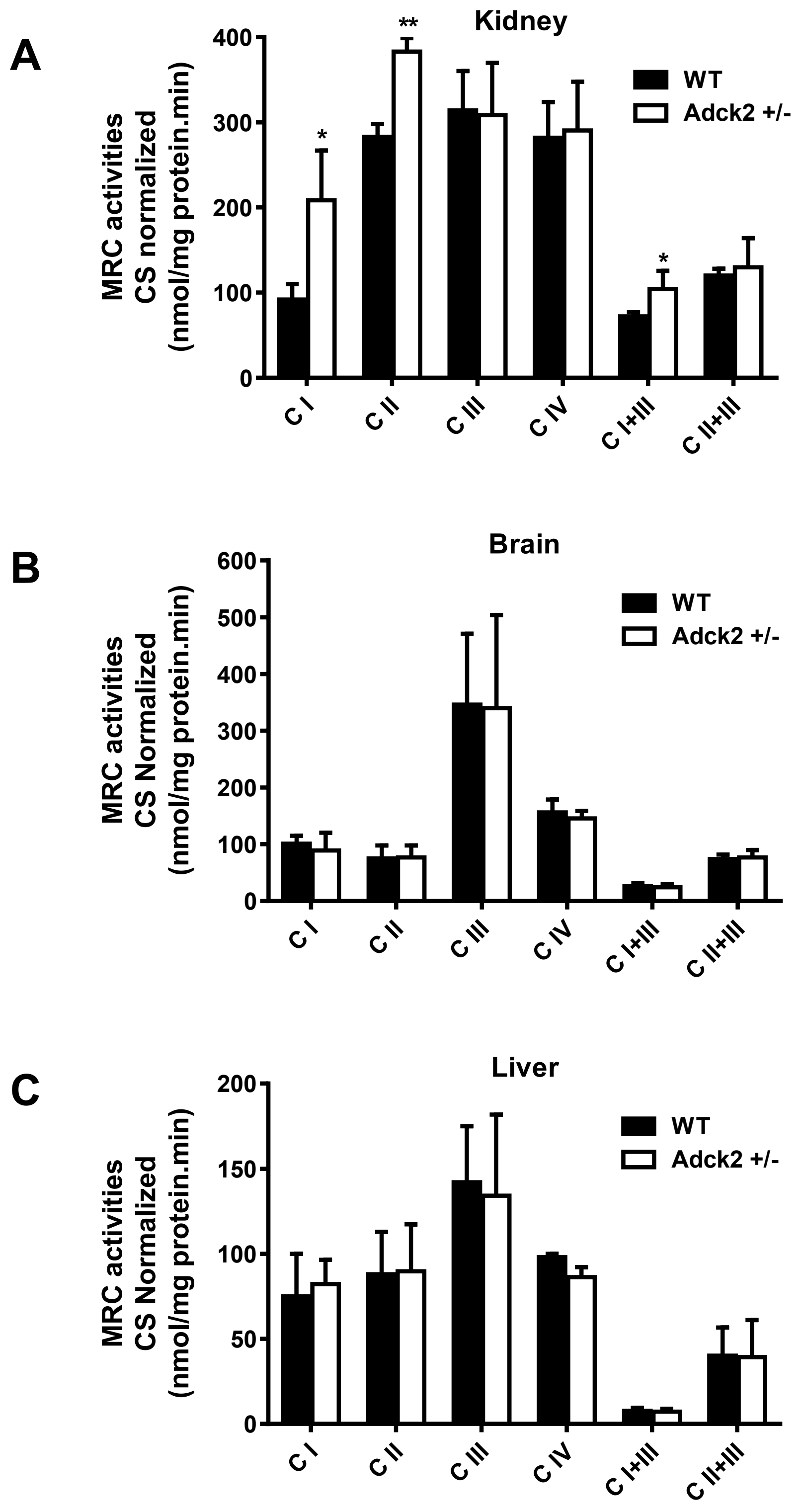

Figure S5

Supplementary Figure 5 related to Fig. 3. *Mitochondria properties of mouse tissues.* Mitochondrial respiratory chain activities normalized to citrate synthase were determined in kidney (A), brain (B), and liver (C) of *Adck2+/-* and WT mice. Bars represent mean + SD. $*p \leq 0.05$. $**p \leq 0.01$ vs. WT; n=5. Data were analyzed by Student's t test.

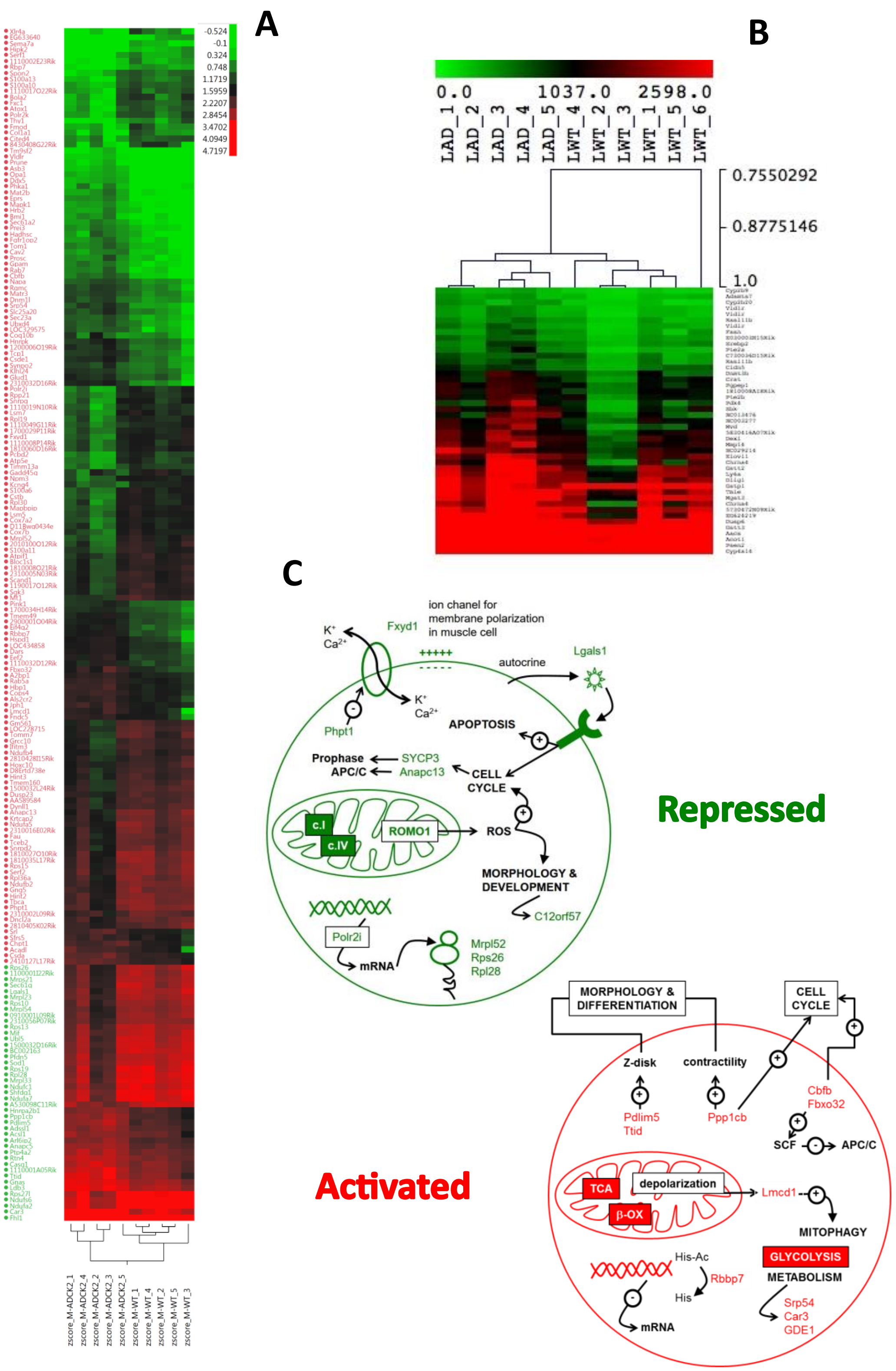

Figure S6

Supplementary Figure 6 related to Fig.5. Microarray analysis in mouse skeletal muscle and liver. Gene expression profile of skeletal muscle (A) and liver (B) from *Adck2*+/- mice compared to aged-matched WT animals. C. Scheme of cellular pathways affected by the mutation based on microarray results from muscle (Supplementary Table 1).
